# Machine learning-based morphological brain analysis in schizophrenia and unaffected siblings: a multisite study of potential risk markers

**DOI:** 10.3389/fnins.2026.1688282

**Published:** 2026-05-20

**Authors:** Ishida Manabu, Kenji Tanigaki, Nobuhiro Ogawa, Naoki Nitta, Akihiko Shiino

**Affiliations:** 1 Department of Neurology, Shimane University, Matsue, Shimane, Japan; 2 Research Institute, Shiga Medical Center, Moriyama, Shiga, Japan; 3 Department of Neurology, Shiga University of Medical Science, Otsu, Shiga, Japan; 4 Department of Neurosurgery, Shiga University of Medical Science, Otsu, Shiga, Japan; 5 Molecular Neuroscience Research Center, Shiga University of Medical Science, Otsu, Shiga, Japan

**Keywords:** schizophrenia, brain morphometry, machine learning, magnetic resonance imaging, siblings, ventral striatum, reward circuit, voxel-based morphometry

## Abstract

**Background and hypothesis:**

Assessing schizophrenia risk factors is crucial for developing early preventive interventions. We hypothesized that unaffected siblings, who share high genetic risk, exhibit neuroanatomical signatures similar to affected patients, potentially reflecting early pathogenic processes.

**Study design:**

To overcome single-center limitations, we analyzed 1,018 participants from five independent, public databases. Brain MRIs were standardized via voxel-based morphometry, and covariate-adjusted z-scores were calculated for regional volumes. An ensemble support vector machine (SVM) approach, incorporating multiple models to ensure robustness, was employed to extract a multidimensional brain signature, from which a schizophrenia-like score (SPS) was derived.

**Results:**

The ensemble SVM achieved high classification performance (AUC = 0.99861). Across all databases, patients exhibited consistent volume reductions in frontal, temporal, insular, and thalamic regions, alongside globus pallidus enlargement. Notably, unaffected siblings were 3.8 times more likely to show brain morphological similarities to patients than were healthy controls. Furthermore, we identified a novel imaging phenotype in siblings: increased ventral striatal volume, which positively correlated with the SPS. This feature, absent in established schizophrenia, suggests a potential compensatory mechanism or a transient developmental marker of risk.

**Conclusion:**

Applying machine learning to large-scale, multi-site neuroimaging data effectively identifies structural endophenotypes. Our findings highlight unique structural characteristics, specifically the enlarged ventral striatum, as a critical biological metric for identifying high-risk individuals before clinical onset.

## Introduction

Schizophrenia is a complex psychiatric disorder characterized by a broad spectrum of symptoms, including positive and negative symptoms, as well as cognitive impairments in memory and attention. Recent large-scale meta-analyses and mega-analyses of structural MRI involving thousands of individuals have robustly demonstrated reduced brain volumes—particularly in the amygdala, hippocampus, thalamus, and cortical lobes—and altered structural connectivity relative to healthy controls (Haijma et al., 2013; van Erp et al., 2016; van Erp et al., 2018; Serdarevic et al., 2018; Serdarevic et al., 2018). While some of these structural differences likely present pre-morbidly, ongoing volume reductions from the at-risk state to the first episode of psychosis are often associated with poorer clinical outcomes (Fusar-Poli et al., 2013; van Haren et al., 2008). Furthermore, post-mortem studies provide pathological validity to these findings, pointing to a multifactorial pathophysiology of reduced neuronal size and dendritic arborization (Rajkowska et al., 1998; Glantz and Lewis, 2000). Recent large-scale studies from the ENIGMA Schizophrenia Working Group have further specified these changes, demonstrating that temporal cortex alterations are already evident in individuals at-risk mental state (ARMS) and are significantly associated with the severity of formal thought disorders and language impairments (ENIGMA Clinical High Risk for Psychosis Working Group et al., 2021).

Despite these insights, identifying robust and generalizable biomarkers for individual diagnosis remains a significant challenge. Many findings in observational epidemiology remain only suggestive or weak, and results are frequently confounded by long-term antipsychotic treatment and the high clinical heterogeneity of the disorder (Fusar-Poli et al., 2013; Kraguljac et al., 2021). To address these limitations and distinguish core neurobiological features from secondary effects, researchers have turned to unaffected siblings of patients. Given that siblings share a significant genetic liability—with a reported risk increase of approximately 6–10 times (Chou et al., 2017; Lichtenstein et al., 2006)—their brain morphology may represent an endophenotype reflecting underlying genetic risk before the onset of clinical symptoms (Prasad and Keshavan, 2008).

Recent frameworks have leveraged the power of ensemble deep learning combined with orthogonal linear transformations. By reducing the dimensionality of structural MRI (sMRI) data and utilizing a voting-based ensemble of models like Visual Geometry Group Net (VGGNet) and Residual Network (ResNet,) researchers have achieved enhanced detection performance and better generalization (Varaprasad et al., 2026; Sarveswaran and Rajangam, 2025; Sarveswaran and Rajangam, 2025). In addition to pure accuracy, explainability has become a focal point in psychiatric artificial intelligence (AI). To distinguish between early-onset schizophrenia (EOS) and bipolar disorder (EBD), researchers have utilized region-of-interest (ROI) -based brain surface morphology parameters, including cortical thickness and fractal dimension. The use of SHAP (SHapley Additive exPlanations) alongside algorithms like AdaBoost allows for the identification of specific biological markers that drive the differential diagnosis (Saglam et al., 2023). Furthermore, the fusion of diverse data domains has pushed the limits of diagnostic precision. By combining spatial domain features (e.g., texture analysis via Local Binary Patterns) with frequency domain features (e.g., spectral analysis via Fast Fourier Transform), AI-driven approaches can capture a more comprehensive representation of neural abnormalities. Such hybrid feature fusion techniques have demonstrated high accuracy levels exceeding 90%, reflecting their potential for robust clinical classification (Sun et al., 2021), (Tyagi et al., 2025).

While structural imaging has revealed various morphological changes in the schizophrenic brain, conventional univariate statistical methods often fail to capture the multifaceted nature of brain abnormalities at an individual level. To address these limitations, recent research has shifted toward advanced machine learning (ML) frameworks that can integrate complex, high-dimensional data (Zarogianni et al., 2013; Oh et al., 2020). In this context, choosing an appropriate algorithm is critical; while deep learning offers high performance, simpler yet robust linear models like support vector machines (SVM) are often preferred for their ability to handle high-dimensional ROI data while minimizing the risk of overfitting due to multicollinearity (Lee et al., 2021).

In this study, we aimed to develop a Schizophrenia-like Score (SPS) by applying an SVM-based ML model to a large-scale dataset of over 1,000 individuals across five independent databases. By leveraging the robust regularization properties of SVM, we sought to capture a broad spectrum of neuroanatomical information from numerous ROIs, ensuring a stable and generalizable indicator of structural similarity to the schizophrenia phenotype. We propose this simplified framework to quantify the complex structural variations observed in schizophrenia and high-risk individuals. If the SPS effectively reflects the neuroanatomical endophenotype, it could serve as a valuable biomarker for evaluating whether genetically high-risk individuals, such as siblings, exhibit intermediate structural signatures, thereby clarifying the link between brain morphology and genetic liability.

## Methods

### Dataset of participants

This study was performed on the clinical information of 1,068 participants with permission from SchizConnect^1^. Data were drawn from five databases—NUSDAST (Kogan et al., 2016), NMorphCH (Alpert et al., 2016), MCICShare (Gollub et al., 2013), BrainGluSchi (Bustillo et al., 2017), and COBRE (Chyzhyk et al., 2015) —and included 3D brain MR images at the first visit of 1,027 participants. Nine subjects were excluded from subsequent analyses due to poor image quality (Image Quality Ratio [IQR] < 70%). The demographic features of the participants are summarized in Table 1. The final sample of 1,018 subjects comprised 488 healthy controls, 475 patients with schizophrenia, 12 with schizoaffective disorder, nine with bipolar disorder, and 34 siblings of patients with schizophrenia. In this study, “schizophrenia” includes both strict and broad cases. For machine learning (ML), 80% of healthy participants and 80% of patients with schizophrenia from BrainGluSchi, MCICShare, NMorphCH, and NUSDAST were randomly selected as training data, with the remaining 20% used as validation data. The COBRE database, which included bipolar and schizoaffective cases, was used as test data to evaluate model performance.

**Table 1:** Summary of participants in each database.

Site	NUSDAST		NMorphCH		MCICShare		BrainGluSchi		COBRE	
Total	325		82		204		180		172	
Diagnosis	Sz	NC	Sz	NC	Sz	NC	Sz	NC	Sz	NC
No. of participants	141	184	58	24	109	95	89	91	78	94
Age (SD)	34.7(12.8)	29.7(13.5)	32.8(7.3)	24.7(2.9)	34.3(11.1)	33.3(12.2)	35.9(13.9)	37.5(12.4)	37.1(13.8)	37.8(11.7)
Sex (F/M)	51/90	99/85	18/40	14/10	26/83	30/65	11/78	27/64	15/63	27/67
TIV (SD)	1,450 (157)		1,442 (145)		1,491 (141)^b^		1,481 (135)^b^		1,470 (147)	
	1,422 (161)^a^	1,471(152)^a^	1,440 (136)	1,448 (168)	1469 (151)^a^	1,516(124)^a^	1,492 (139)	1,469(131)	1,468 (146)	1,471(48)
IQR (SD)	80.4 (3.7)	81.1(3.3)	85.6(2.3)	86.0(1.5)	85.7(1.6)	85.8(1.4)	85.9(1.1)	86.2(0.6)	85.8(1.1)	86.2(0.5)
Other	Sibling of Sz = 34		Schizoaffective = 1		–		–		Bipolar = 9 Schizoaffective = 11	

^a^TIV was significantly smaller in patients with schizophrenia than in healthy subjects in NUSDAST (*p* = 0.083, Mann–Whitney *U* test) and MCICShare (*p* = 0.0157, Mann–Whitney *U* test).

^b^TIV of BrainGluSchi and MCICShare participants was significantly greater than that of NMorphCH and NUSDAST participants, respectively (Student’s *t*-test).

Sz, schizophrenia; NC, normal control; TIV, total intracranial volume; IQR, image quality rate.

### MR imaging, voxel-based morphometry, and machine learning

Structural MR images were acquired using a three-dimensional magnetization-prepared rapid gradient-echo (MP-RAGE) sequence. Static field strengths varied by site: 3.0T for BrainGluSchi, COBRE, and NMorphCH; 1.5T for NUSDAST; and either 3.0T or 1.5T for the MCICShare dataset. Imaging resolution was standardized to 1.0 mm^3^ isotropic voxels, except for the MCICShare dataset (0.625 × 0.625 × 1.5 mm). To prepare variables for subsequent machine learning (ML) analysis, MR images underwent preprocessing via voxel-based morphometry (VBM) using BAAD software (version 5.0^2^), as previously described (Syaifullah et al., 2020). This procedure, which incorporates the Computational Anatomy Toolbox (CAT12, version 12.8.2 r1933) within MATLAB R2021a, standardizes brain geometry through global coordinate transformations and corrects local tissue volumes. Specifically, images were normalized to the MNI152NLin2009cAsym space using the geodesic shooting registration algorithm. To preserve the total amount of tissue, the gray matter (GM) segments were modulated by scaling with the Jacobian determinants. These modulated GM maps were subsequently smoothed with an 8 mm FWHM Gaussian kernel. For quality control, we employed the CAT12 automated image quality rating (IQR) and homogeneity checks, excluding datasets with a weighted average quality score below Grade D.

To address the high dimensionality of the imaging data for ML, anatomical regions of interest (ROIs) were defined as the fundamental units of analysis. We identified 290 ROIs using a combination of established atlases: the Automated Anatomical Labeling (AAL) (Tzourio-Mazoyer et al., 2002), Brodmann’s atlas, and the LONI Probabilistic Brain Atlas (LPBA) (Shattuck et al., 2008). These atlases were selected to provide a multi-layered representation of brain structure, incorporating both macro-anatomical landmarks and cytoarchitectonic boundaries. The complete list of the 290 ROIs and their corresponding atlases is provided in Supplementary Table 1. For each ROI, *z*-scores were calculated using the MarsBar toolbox^3^. To ensure biological relevance, total intracranial volume (TIV) and age were included as nuisance variables in the adjustment process. All 290 ROIs were retained in the final model to preserve global and asymmetrical morphometric patterns, with potential multicollinearity addressed via Elastic Net regularization (see Supplementary Table 1 for VIF and Main Effect values).

The SVM with a radial basis function (RBF) kernel was employed for classification. For the underlying standardization process, the BAAD software utilizes a reference sample of 1,031 individuals from the Healthy Brain Network (HBN) and IXI cohorts^4^ to ensure anatomical consistency across datasets. The dataset, consisting of healthy participants and patients with schizophrenia, was partitioned into training and validation sets at an 8:2 ratio. To ensure model stability and mitigate overfitting, multiple partitions were evaluated, and the model yielding the minimum discrepancy in accuracy between the training and validation sets was selected. The SVM outputs were quantified as the “Schizophrenia-like Score” (SPS), representing the distance of a data point from the decision hyperplane and is obtained from the posterior probability function 𝑃𝑟 = (𝑌 = 𝑘|𝑋 = 𝑥), where Y represents class k given input variable x . This probability is transformed via a sigmoid function to constrain the values within the range of [0, 1]. A higher SPS indicates greater structural similarity to the schizophrenia group.

To enhance the robustness and generalizability of the SPS across diverse clinical settings, we implemented an ensemble averaging approach, a strategy recognized for mitigating the fundamental limitations of individual classifiers. Rather than relying on a single SVM model, which may succumb to the “pitfalls” of site-specific noise and overfitting in high-dimensional neuroimaging data (Arbabshirani et al., 2017), the final SPS for each individual was derived by averaging the probability outputs from five distinct SVM models generated during the Leave-One-Database-Out Cross-Validation (LODO-CV).

**Figure 1:**
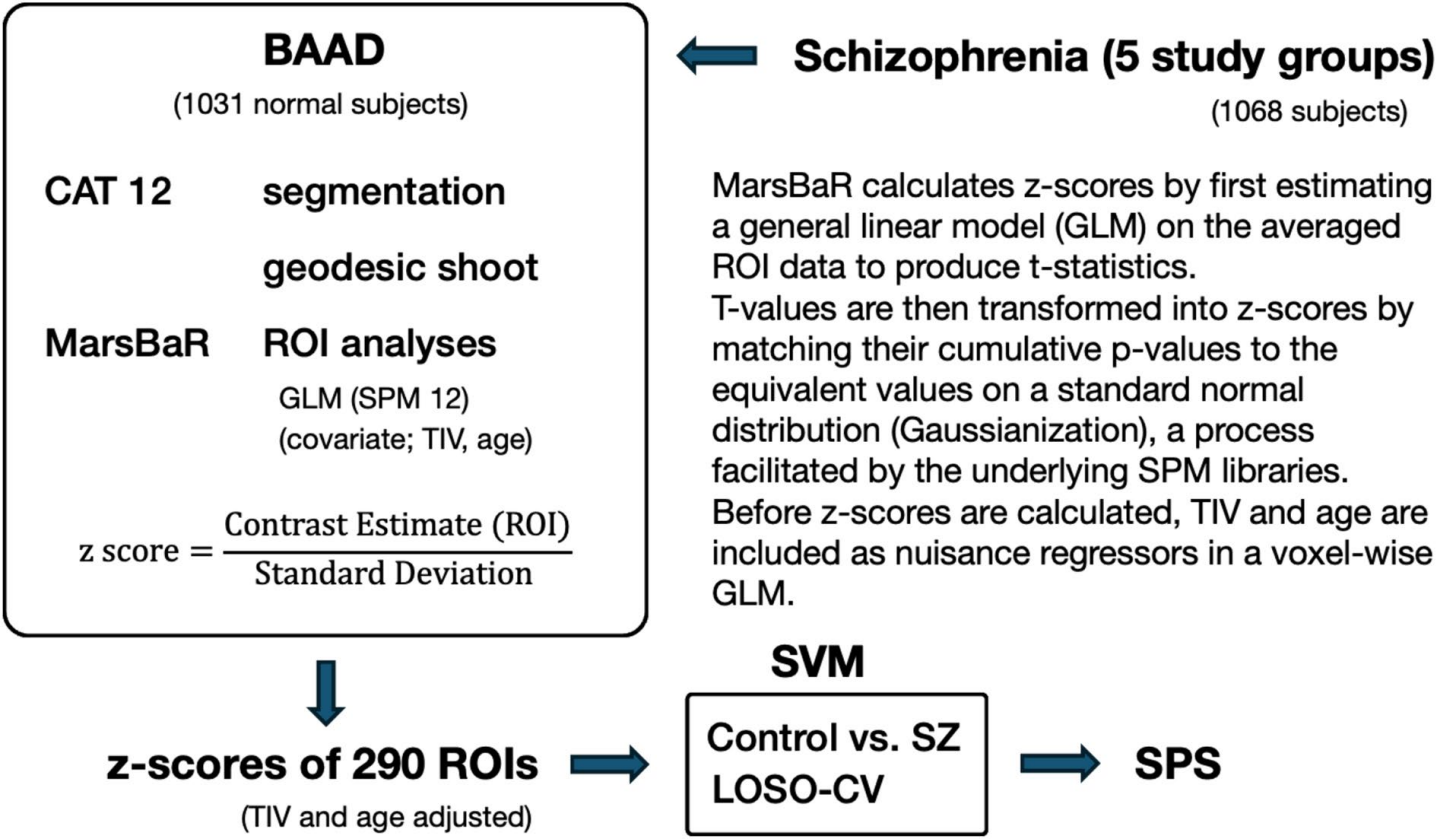
Schematic diagram of the schizophrenia classification process.

This ensemble integration addresses the statistical and computational challenges of single-site models by neutralizing facility-specific biases, such as idiosyncratic imaging protocols or demographic variances. By synthesizing predictions from models trained on heterogeneous data combinations, the resulting ensemble SPS provides a more stable and reliable diagnostic estimate for unseen patient cohorts. Further details of the SVM implementation are described elsewhere (Syaifullah et al., 2020), and the overall workflow is outlined in Figure 1.

For voxel-level analysis, the Computational Anatomy Toolbox 12^5^ was used. Statistical significance was assessed using threshold-free cluster enhancement (TFCE) with 2,000 permutations for each database. Clusters with a family-wise error (FWE) correction were identified. Group-level statistical analyses and figure preparation were performed using JMP Pro^®^ (version 19.0.4). For all inferential statistics, a *p*-value < 0.05 was defined as the threshold for significance.

To address potential overfitting due to multicollinearity among structural features, we initially evaluated a feature reduction approach using Elastic Net to calculate variable importance. However, as this comparison between training and test sets yielded no substantial improvement in generalizability, we ultimately employed an SVM, owing to its superior robustness against multicollinearity in datasets of this scale compared to neural networks or gradient-boosted trees. This advantage stems from the SVM’s objective of maximal margin separation and its adherence to structural risk minimization (SRM), which effectively regularizes the model and prevents overfitting to redundant features. Detailed variable importance results from the Elastic Net analysis are provided in Supplementary Table 1 and Supplementary Figure 2.

In addition to the whole-brain ROI analysis, we focused on the nucleus accumbens as a specific region of interest to investigate structural differences between healthy controls and patient groups. To control for age-related volumetric changes, we extracted a subsample of 400 subjects from the five databases, age- and sex-matched to the 34 siblings of patients with schizophrenia. This subgroup comprised 258 healthy controls, 108 patients with schizophrenia, and 34 siblings. The mean age (±standard deviation) was 22.4 ± 3.5 years for healthy controls, 23.2 ± 2.7 years for patients with schizophrenia, and 21.8 ± 3.6 years for siblings; no significant differences were found in pairwise comparisons among these groups.

## Results

### Brain morphometry of young, healthy subjects from the healthy brain network (HBN) database

To study schizophrenia, which develops during brain maturation, it is essential to collect brain data from healthy children before puberty. Accordingly, we analyzed brain MR images of 2,682 healthy subjects (aged 5–22 years) from the HBN database to evaluate morphological changes associated with brain maturation. Further details regarding the HBN are provided elsewhere^6^.

### Brain morphometric characteristics in schizophrenia

**Figure 2:**
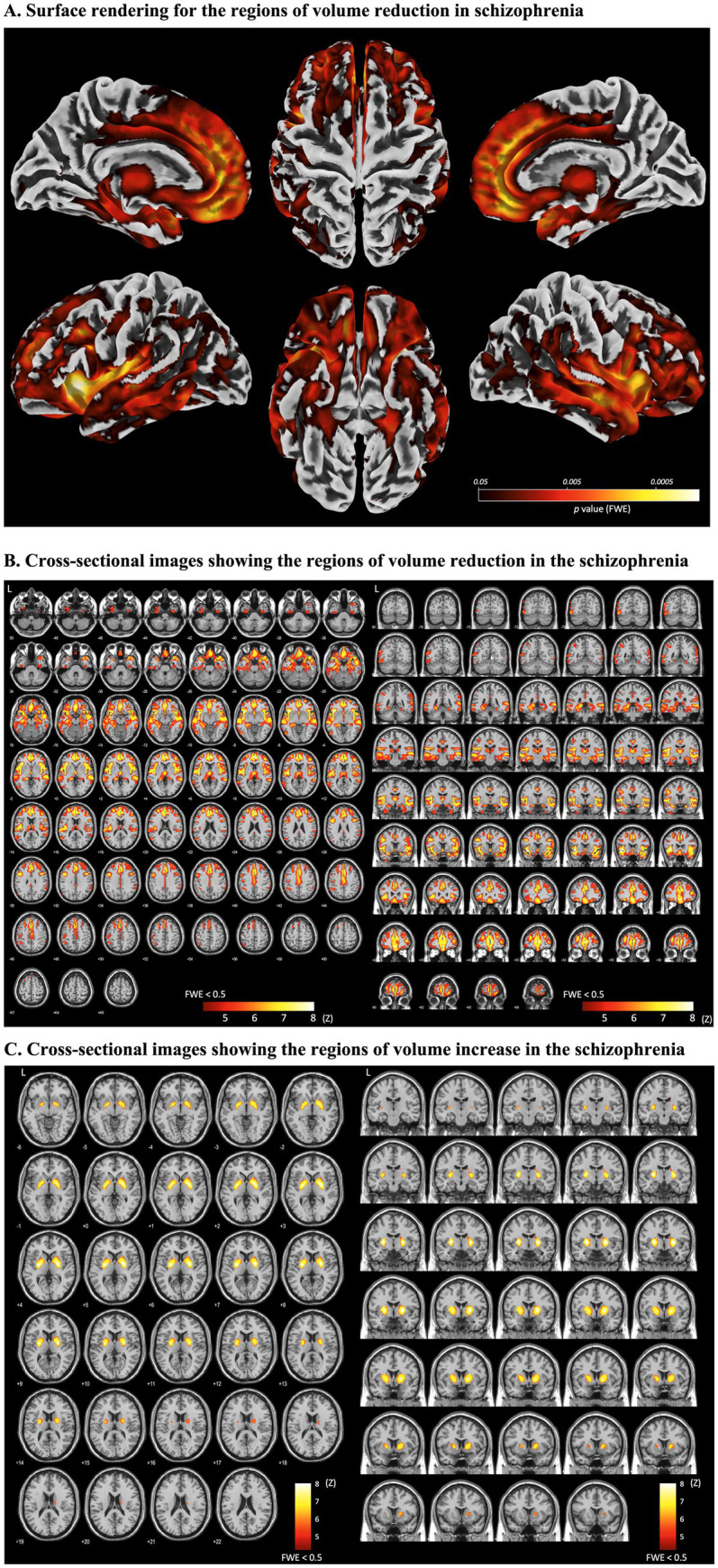
Brain morphological changes in patients with schizophrenia. **(A)** Surface rendering for the regions of volume reduction in schizophrenia. **(B)** Cross-sectional images showing the regions of volume reduction in the schizophrenia. **(C)** Cross-sectional images showing the regions of volume increase in the schizophrenia. **(A)** Surface rendering. Patients with schizophrenia exhibited volume reductions in the frontal, anterior cingulate, temporal, and insular cortices, as well as the orbitofrontal cortex. **(B)** Cross-sectional images of volume reduction. Reductions were observed in the amygdala, hippocampus, and medial thalamus in patients with schizophrenia. **(C)** Cross-sectional images of volume increase. The volume of the globus pallidus and adjacent putamen was increased in patients with schizophrenia. Statistical analysis: Analysis was performed using Random Field Theory (RFT) within Statistical Parametric Mapping (SPM). Areas with a family-wise error (FWE)-corrected *p*-value of less than 0.05 are highlighted. RFT provides a method for adjusting *p*-values across the search volume, performing a function analogous to the Bonferroni correction for multiple individual statistical tests.

We analyzed brain MR images of 488 healthy subjects and 475 patients with schizophrenia from the SchizConnect database to investigate the morphological characteristics of the disease. In the VBM procedure, total intracranial volume (TIV) and age were included as covariates of no interest. Compared to the healthy group, the schizophrenia group exhibited significant volume reductions in multiple regions, including the frontal and temporal lobes, cingulate gyrus, insular cortex, thalamus, hippocampus, and amygdala (FWE, *p* < 0.05) (Figures 2A,B). To evaluate statistical significance, we examined the FDR LogWorth and effect size for each ROI. These effects were particularly pronounced in the prefrontal cortex, insular cortex, middle temporal lobe, and anterior cingulate cortex (Table 2). TFCE confirmed that morphometric changes in schizophrenia were consistent across different databases (Supplementary Figure 3). Furthermore, in patients with schizophrenia, the globus pallidus and adjacent putamen were significantly larger than in healthy controls (FWE, *p* < 0.05) (Figure 2C). The *z*-values for each AAL ROI are summarized in Supplementary Figure 4.

**Table 2:** Results of effect size for disease detection in each ROI (volume/TIV).

MNI ROI name	*p*-value	LogWorth	FDR *P*-value	FDR LogWorth	Effect size
Frontal_Med_Orb_L	2.50E-22	21.6029	2.45E-20	19.6117	0.3054
Frontal_Med_Orb_R	5.16E-21	20.2874	2.53E-19	18.5972	0.2955
Insula_L	8.87E-21	20.0517	2.90E-19	18.5378	0.2937
Rectus_L	1.35E-20	19.8706	3.12E-19	18.5061	0.2923
Temporal_Mid_L	1.90E-20	19.7202	3.12E-19	18.5061	0.2912
Insula_R	1.92E-20	19.7165	3.12E-19	18.5061	0.2912
Rolandic_Oper_L	2.23E-20	19.6523	3.12E-19	18.5061	0.2907
Temporal_Mid_R	4.87E-20	19.3122	5.97E-19	18.2240	0.2881
Cingulum_Ant_L	5.52E-20	19.2585	6.00E-19	18.2214	0.2876
Frontal_Sup_Medial_L	8.27E-20	19.0825	8.10E-19	18.0913	0.2862
Heschl_L	9.67E-20	19.0145	8.62E-19	18.0647	0.2857
Frontal_Sup_Medial_R	2.33E-18	17.6322	1.90E-17	16.7201	0.2746
Frontal_Mid_Orb_L	4.74E-18	17.3247	3.57E-17	16.4474	0.2720
Frontal_Inf_Tri_L	1.25E-17	16.9041	8.73E-17	16.0590	0.2686
Frontal_Inf_Orb_L	1.71E-17	16.7680	1.11E-16	15.9529	0.2674
Temporal_Inf_L	2.22E-17	16.6532	1.36E-16	15.8661	0.2665
Temporal_Sup_R	2.68E-17	16.5718	1.55E-16	15.8101	0.2658
Frontal_Mid_R	5.25E-17	16.2801	2.86E-16	15.5441	0.2633
Frontal_Mid_L	3.26E-16	15.4861	1.68E-15	14.7737	0.2565
Pallidum_L	3.62E-16	15.4411	1.77E-15	14.7532	0.2561

This study was conducted using JMP^®^ software, involving 475 patients with schizophrenia and 488 healthy subjects. LogWorth is a transformation of the *p*-value, defined as -log10(*p*-value), which provides a more visually informative scale for assessing statistical significance—especially for very small *p*-values. A False Discovery Rate (FDR) LogWorth of 2 or higher (corresponding to a p-value of 0.01 or lower) is typically considered statistically significant. Additionally, effect size was used to quantify the magnitude of observed effects, such as differences between group means. The FDR p-values were adjusted using the Benjamini–Hochberg procedure.

### Brain morphometry of young, healthy subjects from the healthy brain network (HBN) database

To study schizophrenia, which develops during the stage of brain maturation, it was necessary to collect data on the brains of healthy children before puberty. Therefore, we analyzed brain MR images of 2,682 healthy subjects aged 5 to 22 years from the Healthy Brain Network (HBN) database to evaluate the morphological changes associated with brain maturation. Details of the HBN are described in detail elsewhere (see Footnote 6).

### Abnormal brain structure in siblings of patients with schizophrenia

**Figure 3:**
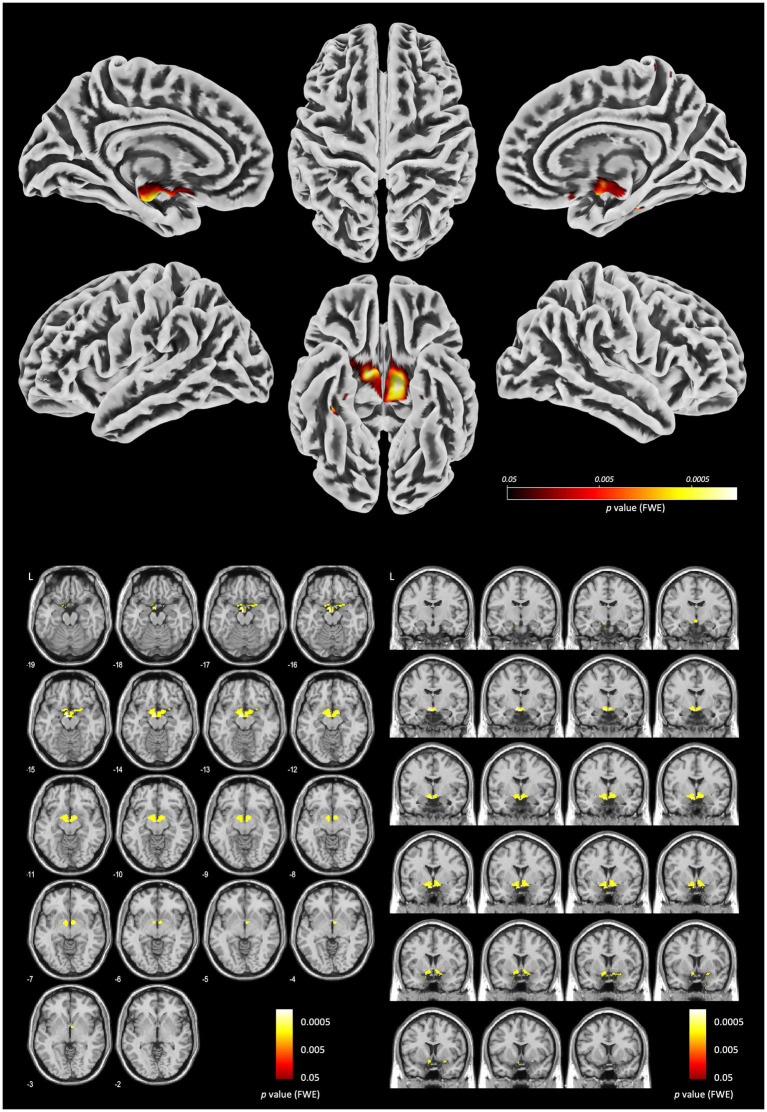
Brain morphological changes in siblings of patients with schizophrenia. (Upper) Surface-based morphometry maps showing regions of significant gray matter volume increases. (Lower) a series of axial and coronal slices with statistical parametric maps (SPM) overlaying the affected areas. Significant clusters are highlighted in a yellow-to-red color scale, corresponding to family-wise error (FWE) corrected p < 0.05 (based on 2,000 permutations). Voxel-based morphometry (VBM) reveals that siblings of patients with schizophrenia exhibit significantly larger gray matter volumes in the bilateral ventral striatum, specifically encompassing the nucleus accumbens, compared to healthy controls. Total intracranial volume (TIV) and age were included as covariates of no interest. While this hyper-morphology in unaffected siblings could be interpreted as a neural correlate of resilience—potentially representing a compensatory response to high genetic load—it may also reflect aberrant neurodevelopmental processes such as impaired synaptic pruning. Its significant correlation with the Schizotypal Personality Questionnaire (SPQ) identifies the ventral striatum as a critical biological metric for assessing psychosis risk across the schizophrenia spectrum.

We analyzed brain MR images of 488 healthy controls and 34 siblings of patients with schizophrenia from the SchizConnect database to investigate morphological characteristics in the sibling group. Total intracranial volume (TIV) and age were included as covariates of no interest. Voxel-based morphometry (VBM) revealed no significant differences in the cerebral cortex. Interestingly, the sibling group exhibited significant enlargement of the ventral striatum (FWE-corrected, *p* < 0.05) (Figure 3), Detailed cluster statistics for this region revealed a significant volume increase (cluster-level FWE corrected 𝑝 < 0.05; cluster size 𝑘 = 1,441 voxels; peak MNI coordinates: 𝑥 = − 6, *y*=8, *𝑧*= − 14; peak 𝑇=7.08; see Supplementary Figure 8 for the full statistical table), a feature not observed in the patients themselves. Based on these findings, we defined a new region of interest (ROI) centered on the areas of the ventral striatum where significant volume differences were detected. This ROI will be utilized for subsequent detailed analyses.

### Differences in brain volume with age in normal individuals, patients with schizophrenia, and their siblings

**Figure 4:**
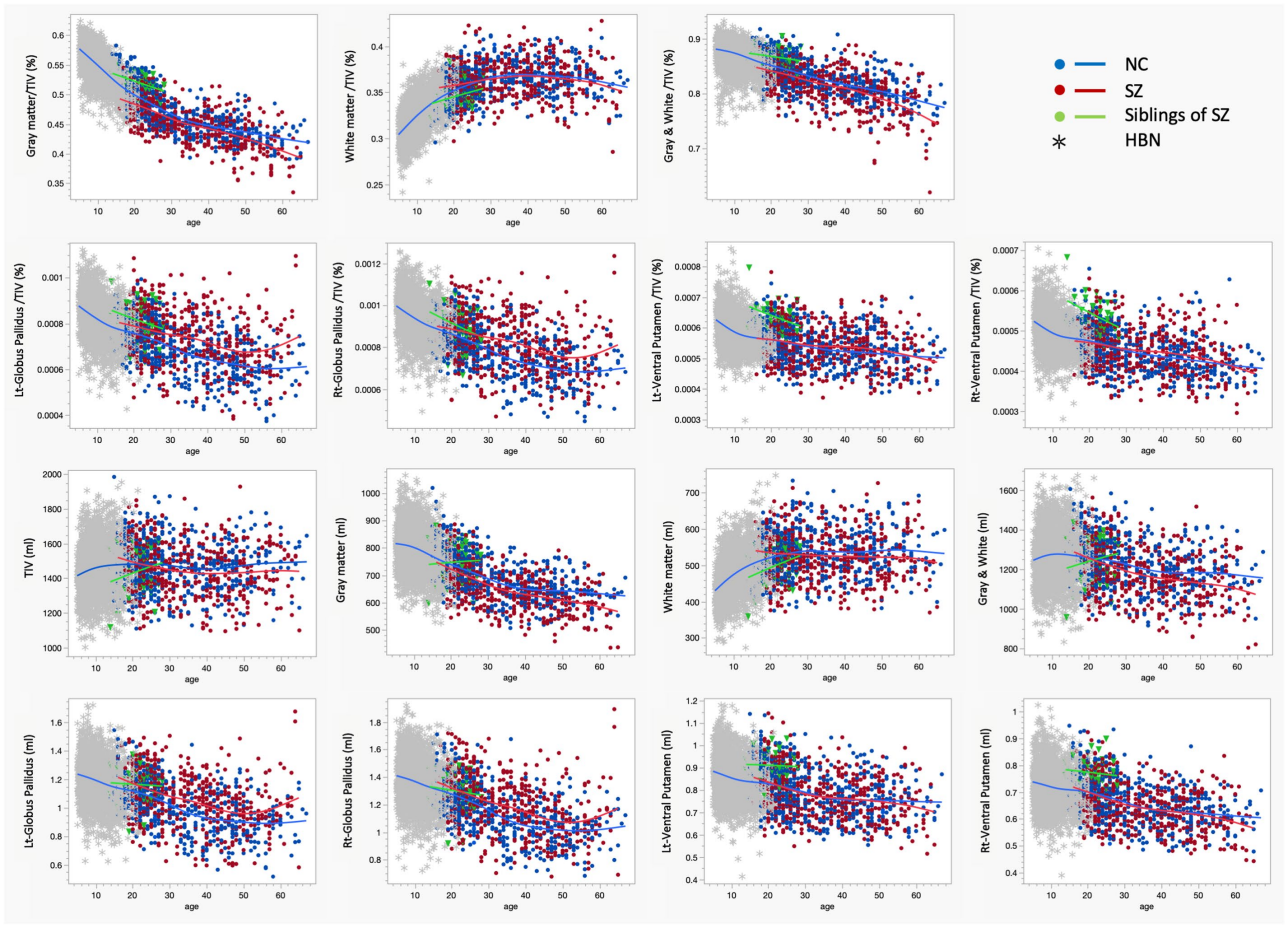
This sixteen-panel scatterplot matrix displays age-related changes in brain volume metrics across four groups: healthy controls (NC, blue), patients with schizophrenia (SZ, red), siblings of patients with schizophrenia (Sibling of SZ, green), and the Healthy Brain Network (HBN, gray). Panels include global measures (gray/white matter) and subcortical structures (globus pallidus, ventral putamen), both in absolute volume (ml) and as a percentage of total intracranial volume. Patients with schizophrenia exhibit smaller gray matter volume than healthy controls across all ages, while white matter volume does not differ significantly. In contrast, siblings of patients with schizophrenia show larger gray matter volume but smaller white matter volume compared to both patients and healthy controls. Consequently, total brain volume is smaller in patients with schizophrenia but larger in siblings, relative to healthy controls. Regarding subcortical structures, both patients and siblings show consistently larger globus pallidus volumes than healthy controls. Notably, while ventral putamen volume does not differ significantly between NC and SZ, it is significantly larger in the sibling group, identifying it as a potential marker for resilience or high genetic risk. NC, healthy controls; SZ, patients with schizophrenia; Sibling of SZ, siblings of patients with schizophrenia; HBN, healthy brain network.

The brain volumes of 488 healthy subjects, 475 patients with schizophrenia, and 34 siblings are plotted against age (Figure 4). We also included data of healthy individuals aged 5–22 years for this study from HBN. In healthy individuals, TIV increases until puberty and subsequently shows minimal fluctuation with age. In contrast, TIV in siblings continues to increase even after puberty, suggesting a delayed maturation pattern. Note that brain volumes were corrected for TIV to minimize the influence of individual differences, particularly sex differences (see Supplementary Figure 5).

In healthy individuals, gray matter (GM) volume decreases with age, while white matter (WM) volume increases until middle age. Patients with schizophrenia exhibited smaller GM volumes than healthy individuals across all age groups, whereas their WM volumes were comparable to those of healthy controls. In siblings, GM volume was larger and WM volume was smaller than in healthy individuals. When combining GM and WM, the total brain volume was smaller in patients with schizophrenia but larger in siblings compared to healthy individuals across all ages.

The volume of the globus pallidus was larger in patients with schizophrenia than in healthy individuals across all ages, whereas no significant difference was observed in the ventral striatum. In siblings, the globus pallidus volume was larger than in healthy controls, mirroring the pattern seen in patients. Conversely, their ventral striatum volume was larger than both other groups. In summary, while siblings show an enlarged globus pallidus similar to patients, their increased ventral striatum volume distinguishes them from the patient group, potentially suggesting a developmental delay in overall brain volume maturation.

### Volumetric changes in the globus pallidus and nucleus accumbens

**Figure 5:**
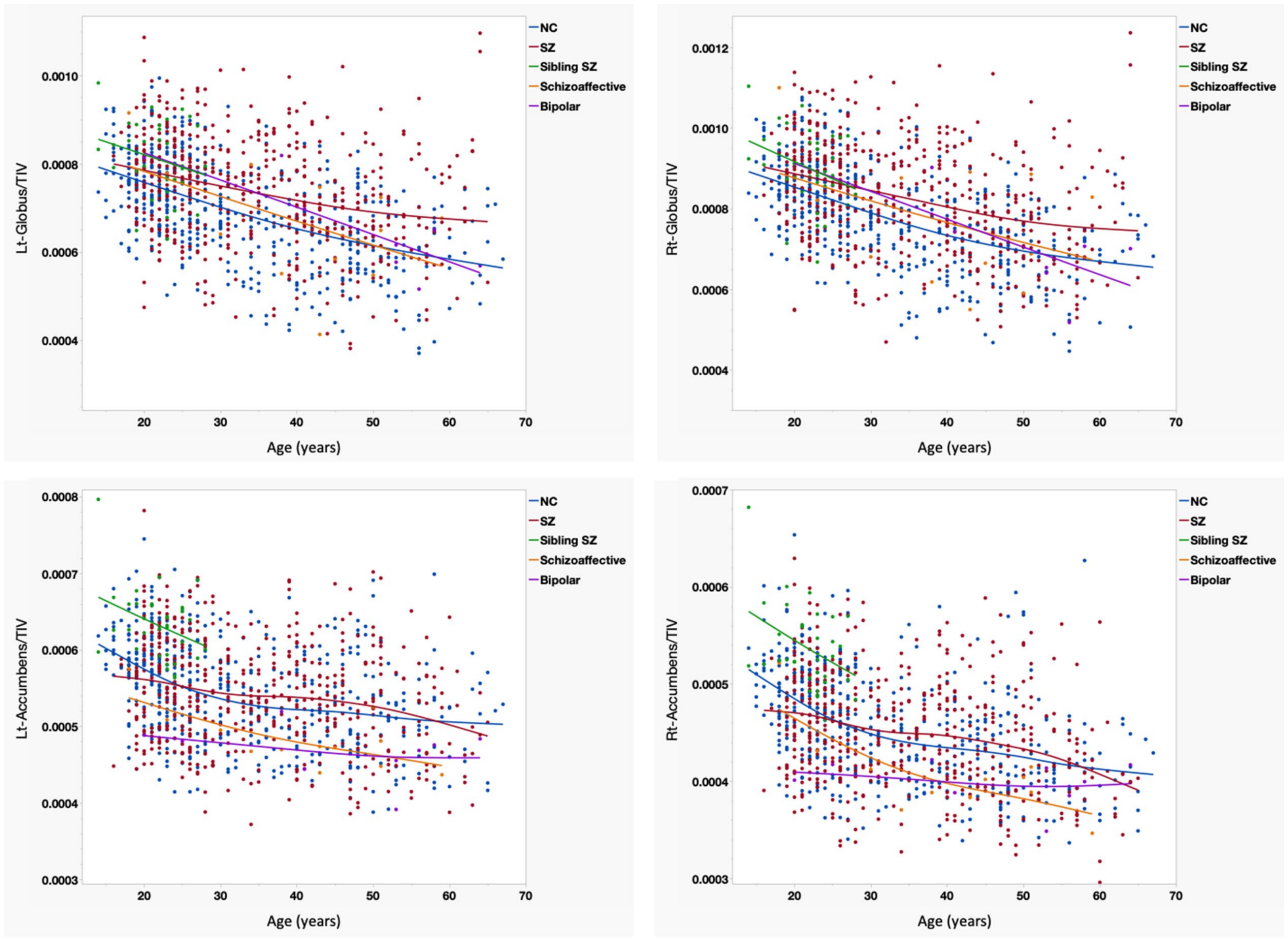
This four-panel scatterplot matrix displays volumetric brain data ratios on the y-axis against age on the x-axis, comparing five groups: healthy controls (NC, blue), schizophrenia (SZ, red), siblings of patients with schizophrenia (Sibling SZ, green), schizoaffective disorder (orange), and bipolar disorder (Bipolar, purple). Trend lines indicate group-specific trajectories across age for the bilateral globus pallidus (upper panels) and nucleus accumbens (lower panels), all adjusted for total intracranial volume (TIV). (1) General trends: All groups demonstrated a consistent age-related decline in volume relative to TIV. (2) Globus pallidus: The SZ group exhibited a distinct trajectory; while NC and other groups showed pronounced age-related reduction, the SZ group maintained relatively higher volumes into older age, suggesting a slower rate of atrophy or compensatory response. (3) Nucleus accumbens: The SZ group showed significantly lower volumes compared to NC in early adulthood (up to age 25), with trajectories converging from the late 20s onwards. In contrast, the bipolar disorder group demonstrated consistently lower volumes across the entire lifespan. (4) Clinical spectrum and resilience: The schizoaffective group followed an intermediate trajectory, reinforcing the concept of a biological continuum. Notably, siblings of SZ patients exhibited higher initial volumes in the nucleus accumbens during youth, potentially reflecting genetic resilience. NC, healthy controls; SZ, schizophrenia; Sibling SZ, siblings of patients with schizophrenia; TIV, total intracranial volume.

Regression analysis of the bilateral globus pallidus and nucleus accumbens revealed distinct developmental and degenerative trajectories across the diagnostic groups. Overall, a consistent age-related decline in volume relative to TIV was observed in both regions across all cohorts, including the healthy controls (Figure 5). In the globus pallidus, the schizophrenia group demonstrated a unique volumetric trajectory; while the healthy controls and other psychiatric groups exhibited a more pronounced age-related reduction in volume, the schizophrenia group maintained relatively higher volumes throughout the lifespan. This trend suggests a potential compensatory volume increase or a markedly slower rate of atrophy in the globus pallidus specifically associated with the pathophysiology of schizophrenia.

Regarding the nucleus accumbens, the schizophrenia group exhibited significantly lower volumes compared to the healthy controls during early adulthood, up to approximately age 25. However, these trajectories converged from the late 20s onwards, with the volumetric gap between the schizophrenia group and healthy controls narrowing with age, suggesting that the neurobiological deficit in the nucleus accumbens in schizophrenia may be a hallmark of the early stages of the illness. In contrast, the bipolar disorder group demonstrated consistently lower volumes in the nucleus accumbens across the entire lifespan compared to the healthy controls. Unlike the converging trend seen in schizophrenia, this persistent reduction in bipolar disorder may represent a stable trait marker or a consequence of chronic neuroprogression related to mood dysregulation. The schizoaffective disorder group generally followed an intermediate trajectory, positioning between the schizophrenia and bipolar disorder groups, further supporting the presence of a biological continuum across these disorders.

**Figure 6:**
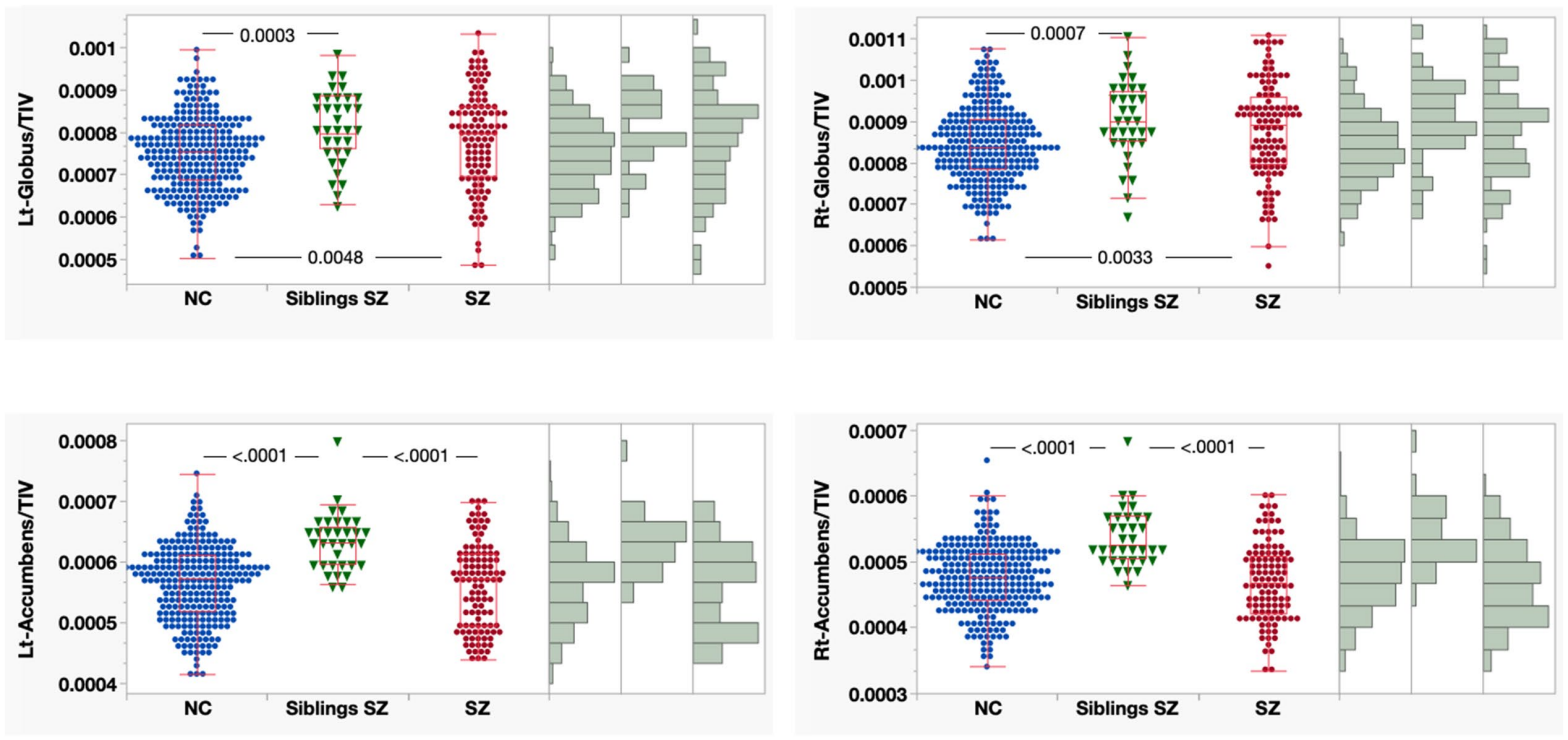
This four-panel figure displays grouped scatter plots with adjacent histograms comparing normalized brain region volumes across three groups: healthy controls (NC, blue circles), siblings of patients with schizophrenia (Siblings SZ, green triangles), and patients with schizophrenia (SZ, red circles). Panels show volumes for the bilateral globus pallidus (upper) and nucleus accumbens (lower), all expressed as ratios to total intracranial volume (TIV). Significant p-values indicating group differences are overlaid on the scatter plots. Regarding subcortical structures, globus pallidus volume was significantly larger in both patients with schizophrenia and their siblings compared to healthy controls. In contrast, nucleus accumbens volume was significantly larger in the sibling group than in both healthy controls and patients with schizophrenia, whereas no significant difference was observed between healthy controls and patients. To adjust for age differences across the groups, a matched subset of 258 healthy controls, 34 siblings, and 108 patients was analyzed. NC, healthy controls; Siblings SZ, siblings of patients with schizophrenia; SZ, patients with schizophrenia; TIV, total intracranial volume.

To further examine these differences while accounting for the effects of aging, we compared volumes across groups after adjusting for three distinct age categories (as presented in Figure 6 and Table 3). This analysis revealed that the nucleus accumbens volume in siblings of patients with schizophrenia was significantly larger than that of both healthy controls and the patients themselves. This enlargement in unaffected siblings may represent a compensatory mechanism that confers resilience against the onset of psychosis, or it could indicate a neurodevelopmental delay characterized by impaired synaptic pruning during adolescence.

**Table 3:** Comparison of nucleus accumbens volumes across groups adjusted for age categories.

ROI	Level		*z*-score	*p*-value	Hodges–Lehmann	Lower CL	Upper CL	Chen’s *d*
Lt-accumbens	Sibling SZ	NC	6.01	<0.0001	0.000064	0.000045	0.000083	1.145
	SZ	NC	−0.53	0.5974	−0.000004	−0.000019	0.000012	−0.054
	SZ	Sibling SZ	−5.02	<0.0001	−0.000070	−0.000096	−0.000044	−1.096
Rt-accumbens	Sibling SZ	NC	6.15	<0.0001	0.000058	0.000041	0.000075	1.225
	SZ	NC	−1.16	0.2458	−0.000007	−0.000020	0.000006	−0.108
	SZ	Sibling SZ	−5.55	<0.0001	−0.000067	−0.000088	−0.000046	−1.194

CL, Confidence Limit.

### Individual distribution of SPS across diagnostic groups and unaffected siblings

**Figure 7:**
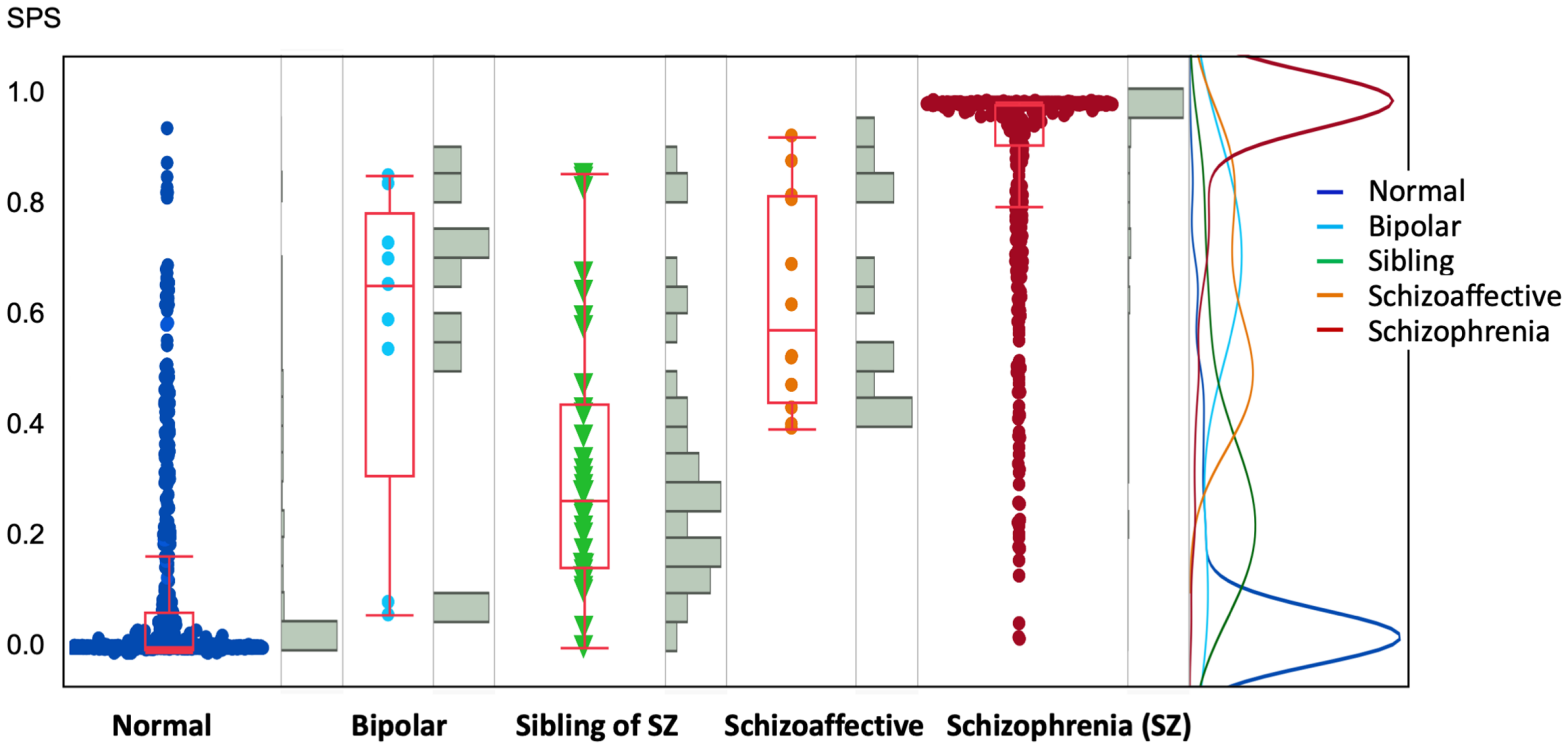
This multi-panel graphic displays SPS scores across five groups: healthy controls (Normal, blue), bipolar disorder (Bipolar, light blue), siblings of patients with schizophrenia (Sibling of SZ, green), schizoaffective disorder (orange), and patients with schizophrenia (SZ, red). Each group is presented with a combination of a box plot, a scatter plot of individual data points, and an adjacent histogram. On the right, a density plot overlays the distributions to highlight group-specific peaks. Higher SPS values indicate greater morphological similarity to the brain structure of individuals with schizophrenia. The mean (SD) SPS was 0.11 (0.18) for healthy controls and 0.99 (0.19) for patients with schizophrenia (SZ). Intermediate scores were observed for schizoaffective disorder (0.63 ± 0.20), bipolar disorder (0.57 ± 0.29), and siblings of SZ patients (0.33 ± 0.24). Histogram analysis revealed distinct peaks at approximately 0.95 for SZ and 0.02 for healthy controls. Notably, among participants aged <30 years, siblings of SZ patients showed a significantly higher risk of having an SPS ≥ 0.5, with an odds ratio of 3.8 and a standardized incidence ratio of 4.5. These findings suggest that unaffected siblings are significantly more likely to exhibit schizophrenia-like structural brain features compared to the general population. SPS, schizophrenia-like score.

We examined the actual distribution of SPS in each individual. Despite the limited sample size, we included cases of bipolar disorder and schizoaffective disorder for reference (Figure 7). SPS represents the morphometric similarity of the brain to that of patients with schizophrenia; a value closer to 1 indicates a higher degree of morphological similarity. While most healthy individuals exhibited low SPS values, most patients with schizophrenia showed high values. Thus, SPS serves as an indicator of the brain morphology characteristic of schizophrenia. Most siblings had SPS values of 0.5 or less; however, a subgroup (*n* = 7/34 = 20.6%) with SPS values exceeding 0.5 was also identified. The incidence of SPS ≥ 0.5 among siblings of patients with schizophrenia corresponded to an odds ratio of 4.8 and a standardized incidence ratio of 4.0. Many patients with bipolar disorder or schizoaffective disorder also showed high SPS values, indicating morphological similarities across these disorders.

### Morphological correlates of SPS and basal ganglia structural alterations

**Figure 8:**
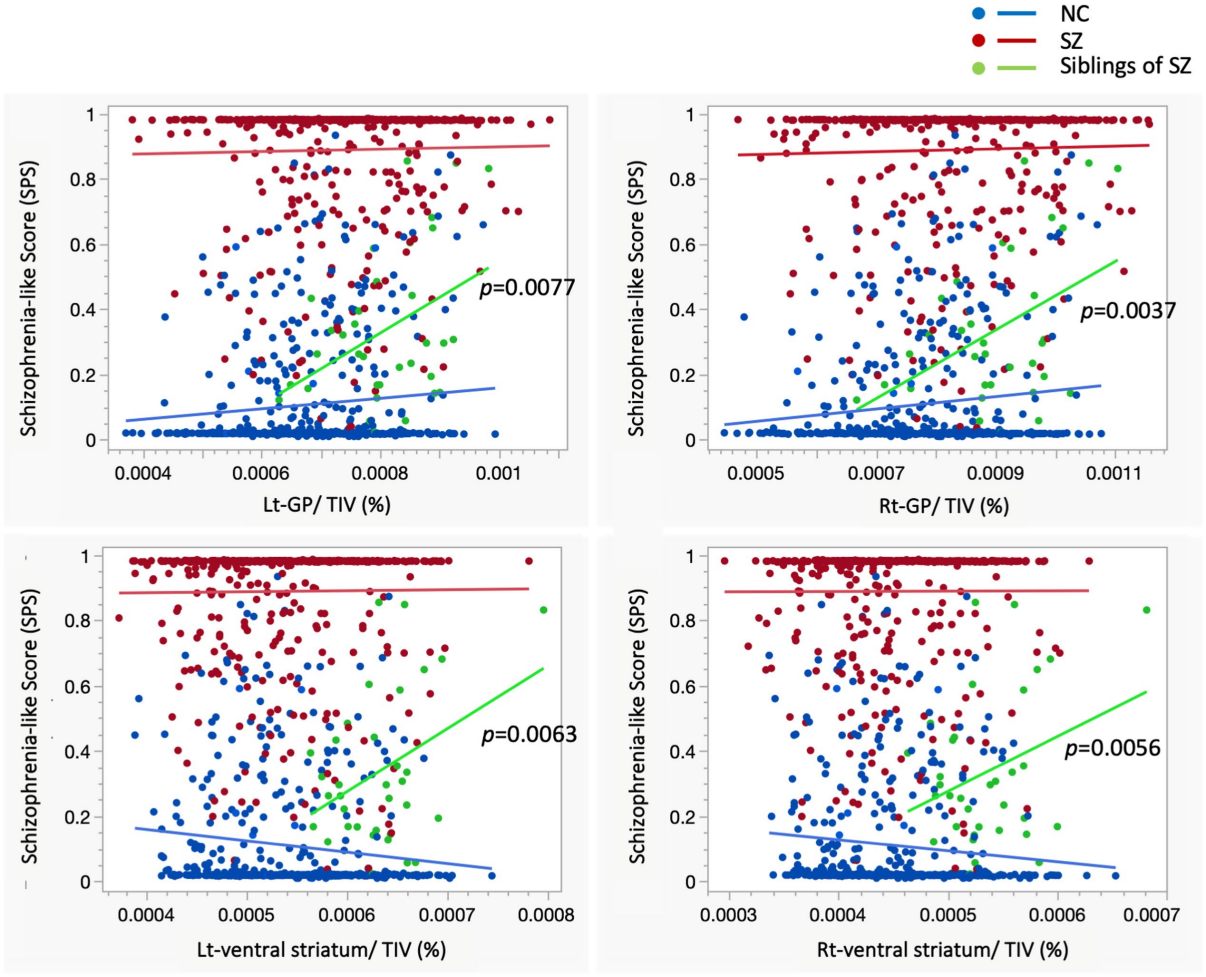
Four scatterplots display volumetric brain data ratios (*x*-axes: Lt-GP/TIV, Rt-GP/TIV, Lt-ventral striatum/TIV, and Rt-ventral striatum/TIV) against schizophrenia-like scores (*y*-axes: SPS) for three groups: healthy controls (NC, blue circles), patients with schizophrenia (SZ, red circles), and siblings of patients with schizophrenia (Siblings of SZ, green circles). Trend lines indicate group-specific correlations, with significant p-values for siblings displayed in each panel. Among siblings, higher SPS scores were significantly associated with larger volumes in both the globus pallidus and ventral striatum. Notably, while the globus pallidus remained enlarged in the SZ group, the ventral striatum volume in patients did not differ from healthy controls. This suggests that the characteristic structural profile of the ventral striatum observed in high-risk siblings may represent a stage-specific marker. The absence of this enlargement in the SZ group could reflect a deviation in neurodevelopmental trajectories or a specific vulnerability of the reward system during the transition from genetic risk to illness onset. NC, healthy controls; SZ, schizophrenia; GP, globus pallidus; TIV, total intracranial volume.

The analysis revealed that increased volumes of the globus pallidus and putamen, along with decreased frontal lobe volume, were significantly associated with SPS. This pattern reflects the morphological features typically observed in schizophrenia, suggesting that basal ganglia alterations in unaffected siblings may serve as a potential endophenotype for disease risk (Table 4). In siblings, larger volumes of the globus pallidus and ventral striatum correlated with higher SPS scores (Figure 8). Since globus pallidus enlargement is a well-established feature of schizophrenia, its association with the SPS is a consistent finding. Similarly, enlargement of the ventral striatum has been suggested to be associated with schizophrenia or an elevated risk of onset. However, as this specific morphological change in the ventral striatum is typically absent in both healthy individuals and patients with established schizophrenia, this feature may diminish with maturation. The observed enlargement of the ventral striatum in siblings—a feature not present in either healthy controls or patients with schizophrenia—presents a complex interpretational challenge. It remains unclear whether this feature represents a transient developmental process that attenuates with maturation or a compensatory mechanism aimed at mitigating genetic vulnerability to schizophrenia.

**Table 4:** Significance test for ROI related to SPS in siblings.

MNI ROI name	*P*-value	LogWorth	FDR *P*-value	FDR LogWorth	Effect size
Globus Pallidus_R	0.00003317	4.4791	0.0034	2.4705	0.3120
Putamen_L	0.00012125	3.9163	0.0045	2.3484	0.2895
Globus Pallidus_L	0.00013187	3.8799	0.0045	2.3484	0.2880
Frontal_Sup_Medial_R	0.00022303	3.6516	0.0057	2.2451	0.2783
Frontal_Med_Orb_R	0.00110132	2.9581	0.0191	1.7196	0.2467
Putamen_R	0.00119913	2.9211	0.0191	1.7195	0.2449
Frontal_Sup_L	0.00248175	2.6052	0.0316	1.4997	0.2290
Frontal_Sup_Orb_R	0.00368504	2.4336	0.0418	1.3792	0.2199
Frontal_Mid_Orb_R	0.00443872	2.3527	0.0453	1.3441	0.2155

Logworth: The quantity -log10(*p*-value). This transformation adjusts *p*-values to provide an appropriate scale for graphing. A value that exceeds 2 is significant at the 0.01 level (−log10(0.01) = 2).

The False Discovery Rate: *p*-value calculated using the Benjamini–Hochberg technique.

### Robust performance and generalizability of the SPS model

The ensemble SVM model used to derive the SPS achieved an overall AUC of 0.9989, with an accuracy of 97.9%, sensitivity of 97.4%, and specificity of 98.4% at an SPS threshold of 0.5 (Table 5). To evaluate the generalizability of the classifier, Leave-One-Database-Out Cross-Validation (LODO-CV) was conducted, demonstrating consistently high performance across all external validation sets (Supplementary Table 2).

**Table 5:** Discrimination accuracy of machine learning per database.

Site	NUSDAST	NMorphCH	MCICShare	BrainGluSchi	COBRE	Total
Num. of participants	330	122	202	176	172	1,002
AUC	0.9979	0.9975	1.0000	0.9953	0.9996	0.9986
Accuracy (%)	97.9	93.1	99.5	95.0	98.8	97.9
Sensitivity (%)	97.3	8400.0	99.1	94.4	97.4	97.4
Specificity (%)	98.4	100.0	100.0	95.6	100.0	98.4
PPV (%)	97.9	100.0	100.0	95.5	100.0	98.4
NPV (%)	97.9	80.9	99.0	94.6	97.9	97.5
F1 (%)	97.6	94.9	99.5	94.9	98.7	97.9
MCC (%)	95.7	824.1	99.0	90.0	97.7	95.9
PLR	60.6	-	-	21.5	-	61.5
NLR	0.0	0.0	0.0	0.0	0.0	0.0

**Figure 9:**
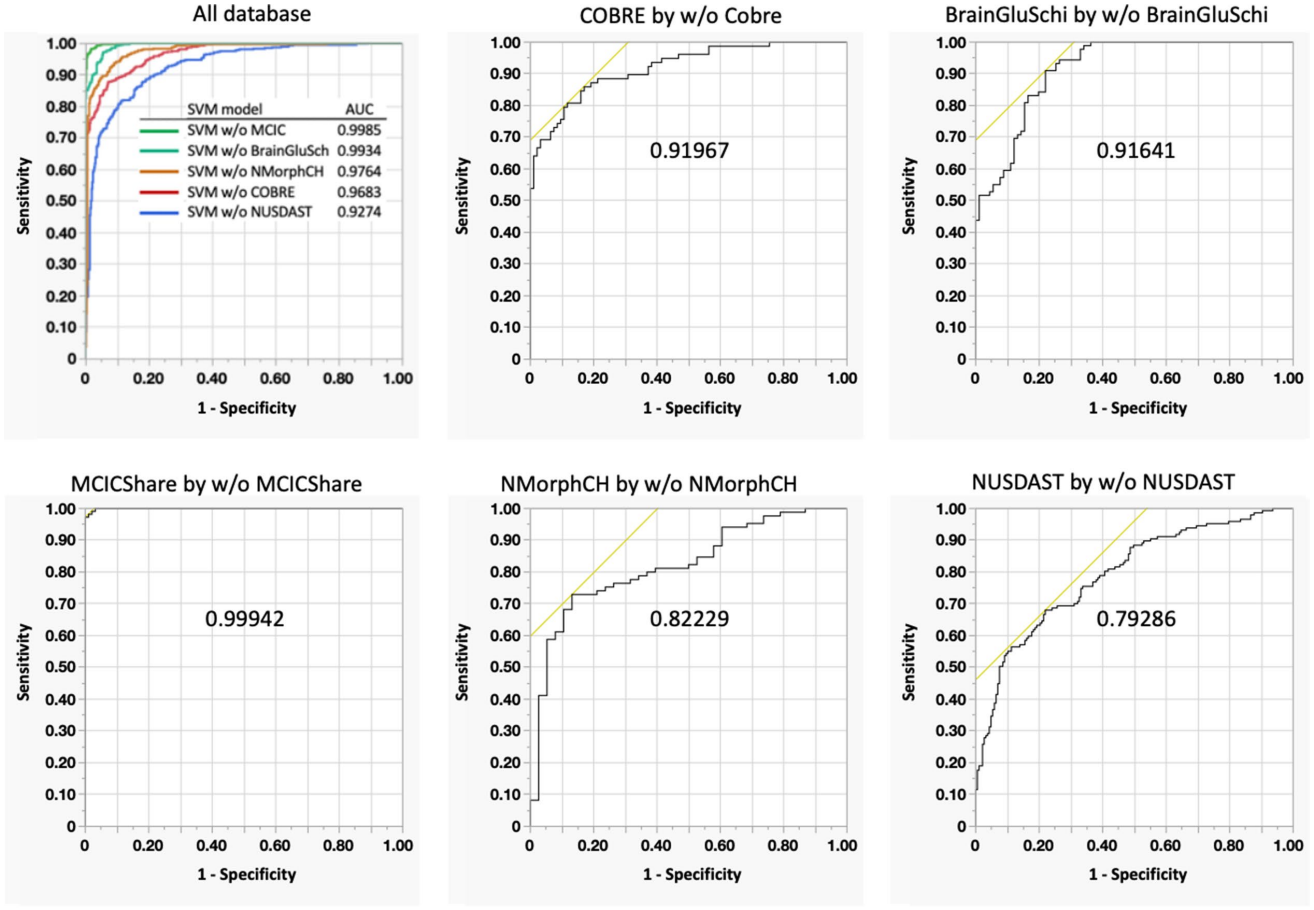
This six-panel graphic displays receiver operating characteristic (ROC) curves for support vector machine (SVM) models. The top-left panel summarizes all models with a legend and color-coded curves (e.g., SVM w/o MCIC in green, SVM w/o COBRE in red). The remaining five panels present individual test datasets (COBRE, BrainGluSchi, MCICShare, NMorphCH, and NUSDAST), each plotting the true positive rate (Sensitivity) against the false positive rate (1 - Specificity). Each plot includes the corresponding Area Under the Curve (AUC) value prominently displayed. LODO-CV results confirm the model’s robustness and generalizability across diverse clinical settings. AUC values remained consistently high, reaching nearperfection for MCICShare (0.999) and maintaining strong performance for COBRE (0.919) and BrainGluSchi (0.916). Even for more challenging datasets such as NUSDAST (0.792) and NMorphCH (0.822), the performance remained within a statistically reliable and clinically relevant range. These findings demonstrate that the model avoids overfitting and reliably predicts outcomes for unseen patient groups. AUC, Area Under the Curve; ROC, Receiver Operating Characteristic; SVM, Support Vector Machine.

The model maintained high AUC values across diverse datasets, specifically 0.999 for MCICShare, 0.919 for COBRE, and 0.916 for BrainGluSchi. Performance remained robust even for more heterogeneous datasets, including NUSDAST (AUC = 0.792) and NMorphCH (AUC = 0.822) (Figure 9). These findings indicate that the model effectively mitigates facility-specific biases and maintains predictive reliability for unseen patient groups across independent clinical settings.

**Figure 10:**
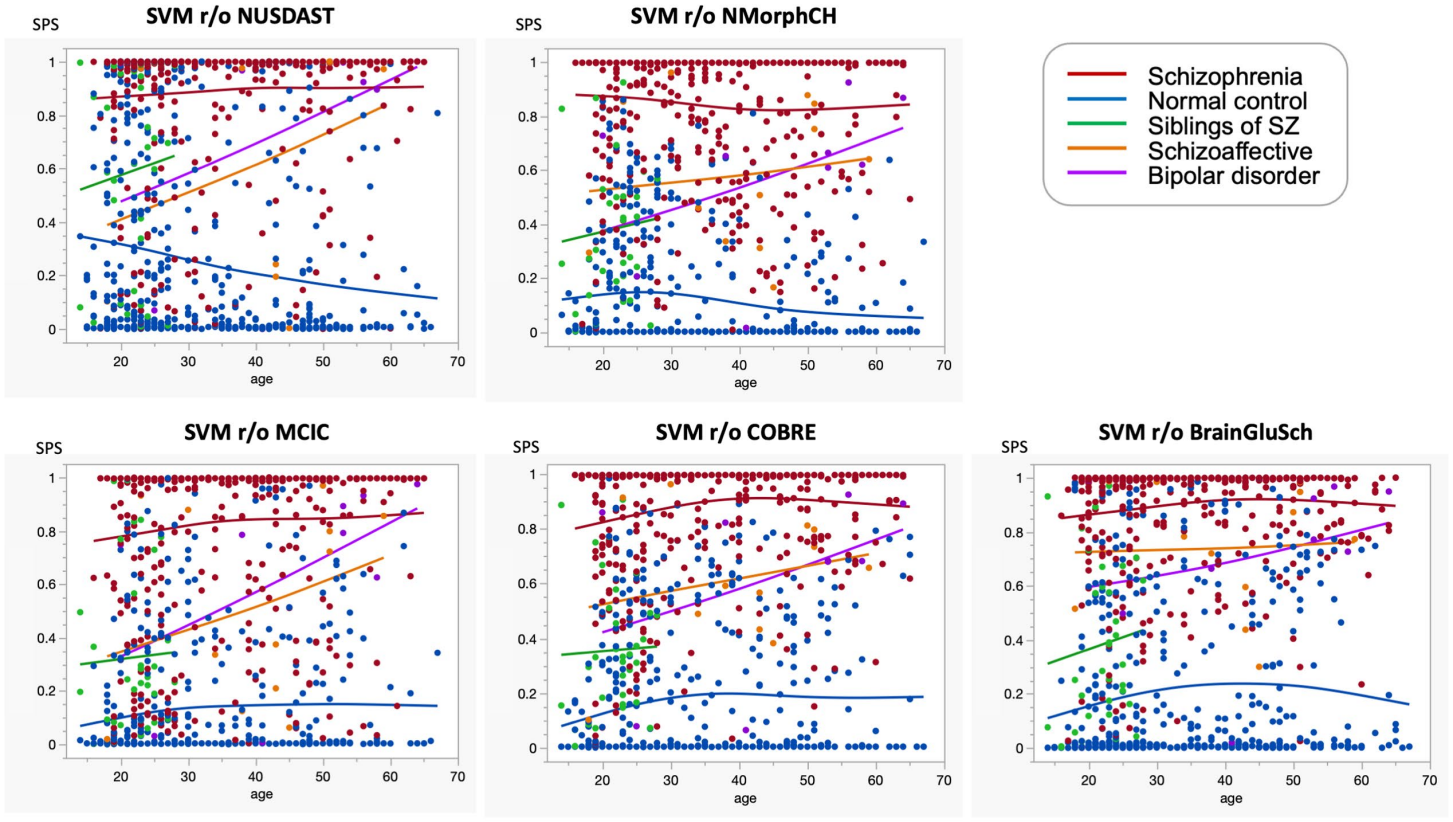
Five scatter plots display schizophrenia-like scores (SPS) on the y-axis versus age on the x-axis, grouped by dataset exclusions in a leave-one-database-out cross-validation (LODO-CV) framework: NUSDAST, NMorphCH, MCIC, COBRE, and BrainGluSchi. Colored dots and trend lines represent five groups: healthy controls (blue), patients with schizophrenia (dark red), siblings of patients with schizophrenia (green), schizoaffective disorder (orange), and bipolar disorder (purple). Each group shows distinct clustering and trend patterns across the age range from approximately 15 to 70 years. Across all five LODO-CV models, a consistent age-dependent increase in SPS was observed not only in siblings but also in patients with schizoaffective and bipolar disorders. This trend suggests that the SPS captures a common neuroprogressive pathway across the psychosis spectrum, where brain structural patterns increasingly converge toward a schizophrenia-like phenotype with advancing age. Notably, this pattern remained robust regardless of which dataset was excluded during model training, confirming the model’s high generalizability. SPS, schizophrenia-like score; NC, healthy controls; SZ, schizophrenia; LODO-CV, leave-one-database-out cross-validation.

Using the five SVM models from the LODO-CV, we evaluated the reproducibility of features associated with siblings and individuals with schizoaffective or bipolar disorders. Although trained solely on healthy controls and patients with schizophrenia, the model effectively identified siblings as an intermediate phenotype. Crucially, across all cross-dataset validations, sibling scores consistently fell between those of controls and patients, regardless of the excluded dataset (Figure 10). This aligns with meta-analytic evidence that structural brain alterations in relatives represent a neuroanatomical endophenotype (Boos et al., 2007; de Zwarte et al., 2019; Zhang et al., 2020; Wang et al., 2024). Furthermore, a consistent age-dependent increase in SPS value was observed among siblings, as well as in patients with schizoaffective and bipolar disorders. This suggests that the SPS captures a common neuroprogressive pathway across the psychosis spectrum, where brain structural patterns increasingly converge toward a schizophrenia-like phenotype with advancing age. This observation supports the concept of accelerated brain aging previously identified via machine learning (Constantinides et al., 2023). Moreover, the model’s ability to capture abnormalities across diagnostic categories aligns with the ‘psychosis continuum’ hypothesis, suggesting shared neurobiological substrates among schizophrenia, bipolar, and schizoaffective disorders (Tamminga et al., 2014; Sorella et al., 2019).

To illustrate the potential clinical utility of the SPS for individual assessment, representative MR images and corresponding scores are presented in Supplementary Figure 6.

## Discussion

The present study demonstrated common morphological brain changes in patients with schizophrenia across cohorts from five distinct databases. Furthermore, siblings of these patients exhibited similarities in frontal lobe and basal ganglia morphology to those seen in schizophrenia, even in the absence of clinical symptoms. Notably, an increased volume of the ventral striatum was observed in siblings—a feature not present in patients with schizophrenia. To the best of our knowledge, this is the first study to report such a finding. The ventral striatum is a key component of reward circuitry, and its developmental immaturity, combined with environmental stress, may play a critical role in disease onset (Ahmed et al., 2015; Salgado and Kaplitt, 2015).

Genetic factors exert a stronger influence on the onset of schizophrenia than environmental factors. For instance, siblings of affected patients have been reported to have a 2.76-fold increased risk in a Danish study (Laursen et al., 2005) and a 7.34-fold risk in a Swedish study (Li et al., 2009). In our study, the odds ratio for siblings to exhibit brain morphology similar to schizophrenia was 4.8, with a standardized incidence ratio of 4.0. Approximately 20% of the siblings in this study already displayed schizophrenia-like morphological changes, suggesting that these alterations precede clinical onset. This evidence of brain abnormalities in siblings, revealed through morphological analysis, underscores the potential for developing risk-assessment tools. Since brain morphological abnormalities in schizophrenia are thought to be multifaceted and associated with both genetic variants and disease risk (Salgado and Kaplitt, 2015), it is highly probable that these morphological similarities, or a high SPS, are closely linked to the risk of developing the disorder.

### Cortical and subcortical abnormalities in schizophrenia

Consistent with previous studies, we demonstrated that patients with schizophrenia exhibit reduced volumes in the cerebral cortex, particularly within the frontal and temporal lobes, the insular cortex, and the limbic system (Schultz et al., 2010; Haukvik et al., 2013). We also observed relatively larger volumes of the globus pallidus in these patients—a finding that may be linked to the risk of disease onset in siblings. This region receives substantial GABAergic input from the striatum and extends axons to neighboring neurons within the globus pallidus.

Morphological brain abnormalities in schizophrenia are prominently localized within the cortico-basal ganglia-thalamus network. Disruption of this circuit is thought to inhibit cortical processing, resulting in cognitive deficits and negative symptoms, while simultaneously promoting subcortical dopamine release, which leads to psychosis (Coyle et al., 2012). Although the enlargement of the globus pallidus remains one of the most enigmatic abnormalities in schizophrenia, it is still unclear whether this represents true hypertrophy or a persistent immature state. As a key component of the cortico-striatal circuit, the globus pallidus plays a unique role in mediating competing inputs through the striatum (Ahmed et al., 2015); however, its precise involvement in the pathophysiology of schizophrenia is not yet fully understood. Dysfunction of the globus pallidus leads to a loss of regulation over excitatory projections from the thalamus to the cerebral cortex (Tarcijonas et al., 2020). Such synaptic dysfunction within neural circuits—particularly those involving thalamocortical projections—may underlie the psychotic symptoms of schizophrenia (Pergola et al., 2015). Notably, our study found that the volume of the globus pallidus was relatively larger in both patients with schizophrenia and their siblings, regardless of age (Figure 3).

### Differential morphological patterns in the ventral striatum

Our study revealed a significant increase in ventral striatum volume specifically among the siblings of patients with schizophrenia—a finding notably absent in the patients themselves. Furthermore, this volume increase was prominent even when compared to the pediatric cohort (aged 5–10 years). While the underlying cause remains to be fully elucidated, this morphological shift in siblings may represent a transient compensatory response to environmental factors or an enhanced neurobiological sensitivity to the environment. Given the cross-sectional nature of our study, it remains unclear whether this finding reflects a truly transient state; this warrants further longitudinal investigation. As the ventral striatum is a core component of the reward circuitry, morphological alterations in this region may be intrinsically linked to the pathophysiology of schizophrenia. Importantly, siblings with larger volumes of the globus pallidus or ventral striatum were more likely to exhibit brain morphology patterns characteristic of schizophrenia (Figure 4).

Increased excitatory transmission from the thalamus to the auditory cortex is hypothesized to underlie auditory verbal hallucinations (Li et al., 2017). The cortico-basal ganglia-thalamus network comprises sensorimotor, associative, and limbic circuits, which undergo significant refinement during brain development (Yin and Knowlton, 2006). Typically, during maturation, gray matter volume (relative to TIV) decreases while white matter volume increases (Tamnes et al., 2017). Our findings indicate that siblings of patients with schizophrenia possess relatively larger gray matter volumes than healthy controls, potentially suggesting delayed brain maturation. This delayed maturation may also be related to the functional dysconnectivity observed in first-episode schizophrenia, where the pallidum exhibits significantly reduced connectivity with regions such as the dorsolateral prefrontal cortex, caudate nucleus, and cerebellum (Tarcijonas et al., 2020).

In the present study, no significant difference was observed in caudate nucleus volume between healthy controls and patients with schizophrenia. This result remained consistent regardless of whether TIV and age were corrected for, or if the analysis was restricted to subjects under 30 years of age. This finding diverges from previous studies, which have reported both enlargement (Hokama et al., 1995; Zhao et al., 2018) and reduction (Venkatasubramanian et al., 2003; Rajarethinam et al., 2007) of the caudate nucleus in schizophrenia.

### Potential utility of machine learning in MRI-based diagnosis

We addressed the clinical implications of the ML paradigm by proposing the automation of brain feature analysis for high-precision diagnostic applications. Utilizing a substantial dataset of over 1,000 individuals, we extracted distinctive morphological features associated with schizophrenia. The generalizability of our SVM model was confirmed through validation on an independent dataset, entirely separate from the training set. Notably, the ensemble SVM yielded an AUC of 0.9986, an accuracy of 97.9% and a precision of 98.4% for the entire dataset. This performance significantly exceeds results previously reported in the literature; for instance, a deep learning study using the COBRE dataset achieved an AUC of 0.846 (Oh et al., 2020), while other approaches using Support Vector Machines (SVM) (Zhu et al., 2022) and maximum uncertainty linear discriminant analysis (de Moura et al., 2018) reported accuracies of 74 and 73%, respectively.

It is often assumed that MRI scans are of limited utility for diagnosing schizophrenia during the first episode or in the prodromal phase. Currently, brain MRI is primarily employed in clinical settings to rule out conditions causing secondary psychosis, such as systemic medical illnesses or substance abuse (Falkenberg et al., 2017; Keshavan et al., 2020). However, our study demonstrates that applying ML to individual-level neuroimaging data enables the highly accurate classification of individuals as either psychotic or healthy. These findings suggest that ML is poised to significantly expand its role in clinical diagnostics (as illustrated by the case examples in Supplementary Figure 6).

### Current landscape of machine learning in schizophrenia structural imaging

The collective findings summarized in Tables 5, 6 illustrate a significant paradigm shift in the application of machine learning to structural MRI in schizophrenia research. Early studies were often constrained by single-site, small-scale datasets, which limited their generalizability and clinical utility (Guo et al., 2025). However, as shown in Table 6, recent advancements in deep learning architectures and the integration of large-scale, multisite consortia such as COBRE and NAPLS have substantially improved diagnostic performance, with AUC values now frequently exceeding 0.85–0.90 (Oh et al., 2020; Zhang et al., 2023). Furthermore, recent architectural innovations utilizing ensemble deep learning and orthogonal transformations have reported classification accuracies reaching as high as 98.9%. In addition to high accuracy, the integration of explainable machine learning and ROI-based surface morphology has proven effective in distinguishing early-onset schizophrenia from other psychiatric conditions, such as bipolar disorder, by identifying specific structural contributors (Saglam et al., 2024). As detailed in Table 6, prognostic models can now identify individuals at clinical high risk (CHR) who are likely to transition to psychosis within 12–24 months with approximately 70–85% accuracy (Koutsouleris et al., 2021; Schmidt et al., 2017). These results suggest that sMRI-derived morphological signatures—particularly in the superior temporal and prefrontal regions—offer a continuous biological metric that may eventually facilitate personalized preventive interventions before clinical onset.

**Table 6:** Summary of machine learning studies using structural MRI for schizophrenia classification.

Study (first author, year)	ML algorithm	Sample size (patients/controls)	Data source	Accuracy/performance (AUC/Acc)	Key findings
Koutsouleris et al. (2021)	Multimodal SVM	334 CHR-P/225 ROD/334 HC	PRONIA	AUC 0.85–0.90	Combining sMRI with clinical data improves prediction for both CHR and Recent-Onset Depression.
Zhu et al. (2024)	XGBoost (ComBat)	705 CHR/460 HC	ENIGMA-CHR	AUC 0.68 convert, 0.73 (CHR vs. HC)	Generalizable predictions using global, multi-center data
Salvador et al. (2017)	SVM/RVM/LDA	65 SCZ/65 HC	Single site (FIDMAG)	Accuracy 74.3 (GM), 70.4(WM)	SVM using sMRI features (GM/WM) provides optimal diagnostic performance compared to other algorithms.
Xiao et al. (2019)	SVM	88 Drug-naïve FES/88 HC	Single site	Accuracy 79.6%	Drug-naïve first-episode schizophrenia (FES) can be identified via regional GM volume changes.
Dluhos et al. (2017)	Meta-model (SVM)	165 SCZ/159 HC	Multisite Utrecht (Cahn) and Prague (Czech Republic)	Accuracy 70–74%	“Meta-model” approach effectively integrates data from different centers to improve generalization.
Sun et al. (2023)	Auto-encoder (Deep Learning)	134 FES/147 HC	Single site	Accuracy 81.3%	Morphological “fingerprinting” via auto-encoders identifies FES with high individual-level precision.
Porter et al. (2023)	Meta-analysis	Systematic Review	154 studies	Multimodal > Single	Multimodal imaging (sMRI + fMRI/DTI) consistently outperforms single modality for classification.
Repple et al. (2023)	SC (Structural Connectivity)	1,114 (SCZ, Bipolar, MDD, HC)	Marburg-Münster	AUC 0.70–0.78	Shared connectivity deficits exist across disorders, but specific patterns distinguish SCZ from Bipolar.
Nieuwenhuis et al. (2012)	SVM	206 SCZ / 228 HC	Two independent samples Utrecht and Madrid	Accuracy 70–71%	Classification accuracy remains stable across independent large-scale cohorts using sMRI.
Pinaya et al. (2019)	Deep Autoencoders	1,113 (SCZ, Bipolar, HC)	Large-scale multi-sample	AUC 0.70–0.75	Deep learning identifies normative deviance in brain structure as a biomarker for SCZ and Bipolar.
Truelove-Hill et al. (2020)	Ridge Regression	1,189 Children/Adolescents	PNC (Philadelphia)	*R*^2^ = 0.81 (Brain Age)	A multidimensional maturation index (NMI) reveals deviant developmental paths in mental disorders.
Schwarz et al. (2019)	Multivariate (Global Pattern)	641 HC, 482 SCZ, 288 BD	IMAGEMEND/KaSP	Reproducible Patterns	Global GM alterations provide a reproducible “biological signature” for both SCZ and Bipolar.
Khuntia et al. (2025)	Data Repository	Clinical High Risk/SCZ	ECNP Networks “Prevention of Psychosis” and “Machine Learning Network	Repository Overview	Establishes an accessible data ecosystem to enhance collaborative ML research in mental health.
Moberget et al. (2019)	Multivariate analysis	1,513 Adolescents	Large-scale sample	Significant Association	Cerebellar gray matter volume is a key indicator of cognitive function and general psychopathology.
Oh et al. (2019)	3D-CNN (Deep Learning)	162 SCZ/148 HC	Multisite (COBRE and NMorph)	Acc 96.4%	Gradient-weighted Class Activation Mapping (Grad-CAM) to visualize regions. 3D-CNN achieved superior accuracy over traditional ML.
Shi et al. (2021)	SVM-RFE	48 SCZ/31 HC	Single site	AUC 0.84 (sMRI) 0.88 (fMRI) 0.94 (Combined)	Combining structural (sMRI) and functional (fMRI) features provides higher sensitivity than single modality.
Zhang et al. (2023)	3D-ResNet (Deep Learning)	1,024 SCZ/1,020 HC	Large-scale (6 datasets)	AUC 0.96 (on-site) 0.88 (external dataset)	Deep learning (ResNet) on 3D sMRI effectively detects SCZ across diverse, large-scale populations.
Wu et al. (2022)	Elastic Net Logistic Regression	57 Female SCZ/50 HC	Single site	AUC 0.90 (sMRI and fMRI, multimodal)	Identified specific structural and functional alterations in female patients, highlighting gender-specific markers.
Hu et al. (2021)	SVM (Multimodal; sMRI and PRS)	390 SCZ/414 HC	Multisite (CHIMGEN)	Acc 75.6% (sMRI) 79.5% (with PRS)	Integrating sMRI with Polygenic Risk Scores (PRS) significantly improved classification over MRI alone.

While most existing risk prediction studies (Table 7) focus on Clinical High-Risk (CHR) or Ultra-High Risk (UHR) individuals defined by attenuated psychotic symptoms, our study investigates unaffected siblings of patients with schizophrenia. Unlike the CHR/UHR criteria, which primarily capture late-stage prodromal symptoms close to clinical onset, the sibling model allows for the identification of stable neurobiological endophenotypes associated with genetic liability, independent of confounding clinical symptoms or medication effects. By identifying neurobiological risk markers in unaffected siblings before any clinical manifestation, it becomes possible to implement proactive environmental modifications. Our machine learning-derived SPS provides a continuous biological metric that can identify a specific subgroup of unaffected siblings who carry an exceptionally high neuroanatomical risk. For siblings exhibiting a high SPS, even in the absence of clinical symptoms, clinician-led proactive environmental modifications—such as stress management and the optimization of social or educational settings—can be implemented more precisely to mitigate the risk of transitioning to a first psychotic episode.

**Table 7:** Summary of MRI-based risk prediction studies for schizophrenia transition.

Author (year)	Method	Sample size (𝑛) (CHR-T/CHR-NT/HC)	Data source	Performance (AUC/Acc)	Key risk prediction findings
Chung et al. (2018)	3D-CNN	455 CHR/210 HC	NAPLS-2 (North America)	CHR to Psychosis AUC: 0.85 (converter vs. non-converter)	Neuroanatomical immaturity captured by 3D-CNN as a transition marker.
Koutsouleris et al. (2016)	VBM	334 participants (177 UHR, 157 HC)	EUFEST	Acc: 67.9% (UHR vs. HC) 70.4% (predicting subsequent onset)	Robust neuroanatomical signatures across international sites.
Cannon et al. (2016)	Multivariate Cox Reg.	596 (UHR-T: 84, UHR-NT: 512)	NAPLS-2 (Multisite)	AUC: 0.71	Clinical risk factors: Unusual thought, suspiciousness, social decline.

Recent large-scale evidence from Omlor et al. (2025) underscores the marked structural brain heterogeneity in schizophrenia, suggesting that illness-related neuroanatomical alterations manifest differently among individuals. This inherent variability aligns with our approach of utilizing the SPS to identify a specific subset of high-risk siblings. Because schizophrenia-related brain alterations do not manifest uniformly across all individuals, it is consistent to assume that only a certain proportion of siblings—those identified by a high SPS—actually harbor the structural signatures that necessitate proactive environmental interventions. Rather than treating all siblings as a uniform group, the SPS captures individualized neuroanatomical risk, enabling targeted environmental modifications for those whose structural profiles align most closely with the heterogeneous signatures of the disease.

The robust performance of our machine learning model aligns with the recent large-scale meta-analysis by Di Camillo et al. (2024), which established benchmark sensitivity and specificity of approximately 80% across 119 neuroimaging studies. Furthermore, the inclusion of rigorous feature selection in our SPS development reflects their findings that methodological refinement significantly enhances the reliability of MRI-based biomarkers. By identifying siblings with a high SPS, we move beyond the diagnostic boundaries explored in their meta-analysis toward a more personalized approach to early risk detection and environmental intervention. While the lack of longitudinal transition data precludes a definitive prognostic evaluation, the identification of a high SPS subgroup among asymptomatic siblings remains clinically significant. As suggested by Guo et al. (2025), brain structural configurations already carry information about individual vulnerability. Therefore, identifying siblings with high structural similarity to patients provides a rationale for pre-emptive environmental modifications to bolster resilience, even before clinical transition is confirmed through long-term follow-up.

## Conclusion

Our study demonstrates that a machine learning paradigm applied to large-scale neuroimaging data enables robust individual-level classification of schizophrenia. By integrating five independent datasets totaling over 1,000 individuals, we developed the SPS, a quantifiable biological marker of brain morphology. Our findings yield two key clinical insights:

First, the SPS achieved an exceptional diagnostic accuracy of 94.5%, significantly surpassing previous models that were limited by single-site, small-scale datasets. Second, and most importantly, we discovered that unaffected siblings of patients exhibit elevated SPS. This suggests that the SPS can capture subclinical neuro-morphological traits associated with genetic liability, even in the absence of clinical symptoms. These results have significant practical implications, providing a metric for both clinical diagnosis and the identification of individuals with high biological risk. While the current study is cross-sectional, the identification of a high-SPS subgroup among siblings provides a rationale for pre-emptive environmental modifications to bolster resilience before symptom onset. For future work, we aim to validate the predictive value of the SPS in longitudinal cohorts to determine its efficacy in forecasting conversion to psychosis. Further integration of multi-modal data, such as genetic and cognitive profiles, may further enhance the precision of this tool in diverse clinical settings.

### Limitations and future work

Our study has several limitations that warrant consideration. First, the sample size of unaffected siblings of patients with schizophrenia was relatively small, which may limit the generalizability of our findings. Second, we were unable to adjust for the potential effects of antipsychotic medications or other therapeutic interventions, which are known to influence brain morphology (Patel et al., 2014). Due to the cross-sectional design of this study, the longitudinal risk of developing schizophrenia in participants with a high SPS remains unknown. While the relationship between the SPS in siblings and the actual risk of transition to psychosis is still a subject of academic debate, our results suggest that the SPS could serve as a valuable metric for early preventive screening. Although these clinical implications cannot be definitively proven through the current data, future longitudinal studies involving larger sibling cohorts will be essential to clarify the score’s prognostic utility.

Additionally, because of the limited number of cases involving bipolar and schizoaffective disorders, we could not develop differentiated algorithms to distinguish schizophrenia from other primary psychotic disorders. Therefore, a high SPS should currently be interpreted alongside other diagnostic methods. Furthermore, we did not apply ComBat GAM-based harmonization to correct for potential site-specific batch effects across the five databases. While ComBat is a standard for multi-site integration, its behavior and validity are technically challenging to maintain when applied to a single, unknown patient in an independent diagnostic setting without re-estimating the model parameters. To ensure clinical translation via a “frozen” diagnostic algorithm, we instead implemented a person-centered standardization strategy using an extensive independent reference database (BAAD, *N* = 1,031) to transform ROI volumes into z -scores. Our preliminary analysis confirmed that this approach effectively preserved biological signals, as the mean effect size (Cohen’s d ) remained highly stable before and after ComBat-GAM correction (0.341 and 0.346, respectively) (Supplementary Figure 7).

Our ultimate objective is to refine this Artificial Intelligence (AI) paradigm to predict disease risk at the pre-onset stage, thereby facilitating early intervention. To address the current lack of generalizability, we aim to contribute to and utilize large-scale, multisite databases to further assess various high-risk clinical populations.

## Data Availability

The raw data supporting the conclusions of this article will be made available by the authors, without undue reservation.

## Footnotes

1
https://schizconnect.org

2
https://www.shiga-med.ac.jp/hqbioph/iBaad/page0

3
https://github.com/marsbartoolbox/marsbar

4
https://brain-development.org/ixi-dataset/

5
https://neuro-jena.github.io/cat/

6
http://fcon_1000.projects.nitrc.org/indi/cmi_healthy_brain_network/Project.html
