# Supplementary material for: Machine learning-based morphological brain analysis in schizophrenia and unaffected siblings: a multisite study of potential risk markers
Source: Front Neurosci. 2026 May 20;20:1688282. doi: 10.3389/fnins.2026.1688282 (PMC13230089; doi:10.3389/fnins.2026.1688282)
Supplement: Supplementary file 1 [file Data_Sheet_1.pdf]

Supplementary materials

Supplementary Figure 1. Conversion of the brain volume into a z-value with correction of the confounding variables

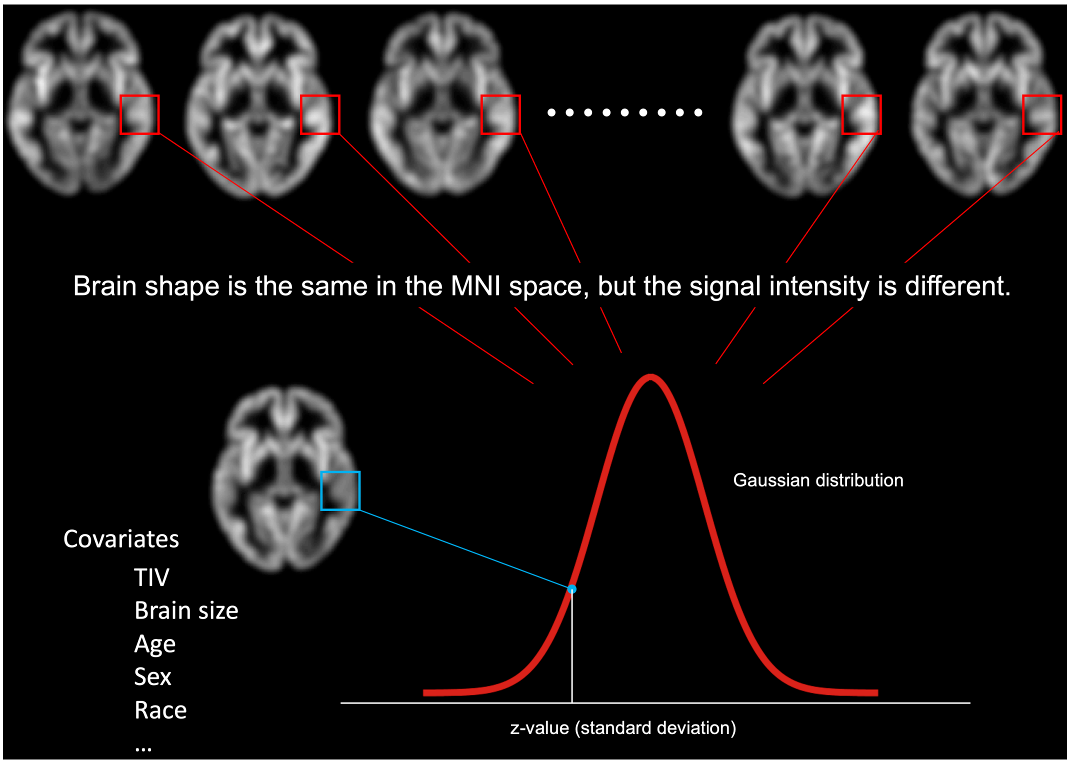

In machine learning, when using data with a small sample size, such as medical data, it is desirable to remove as many confounding variables as possible. In this study, we corrected for total intracranial volume (TIV) and subject age. In the Gaussian distribution, volume can be obtained as a z-value.

Supplementary Figure 2. Regions of significant reduction in TFCE analysis for each of the five databases

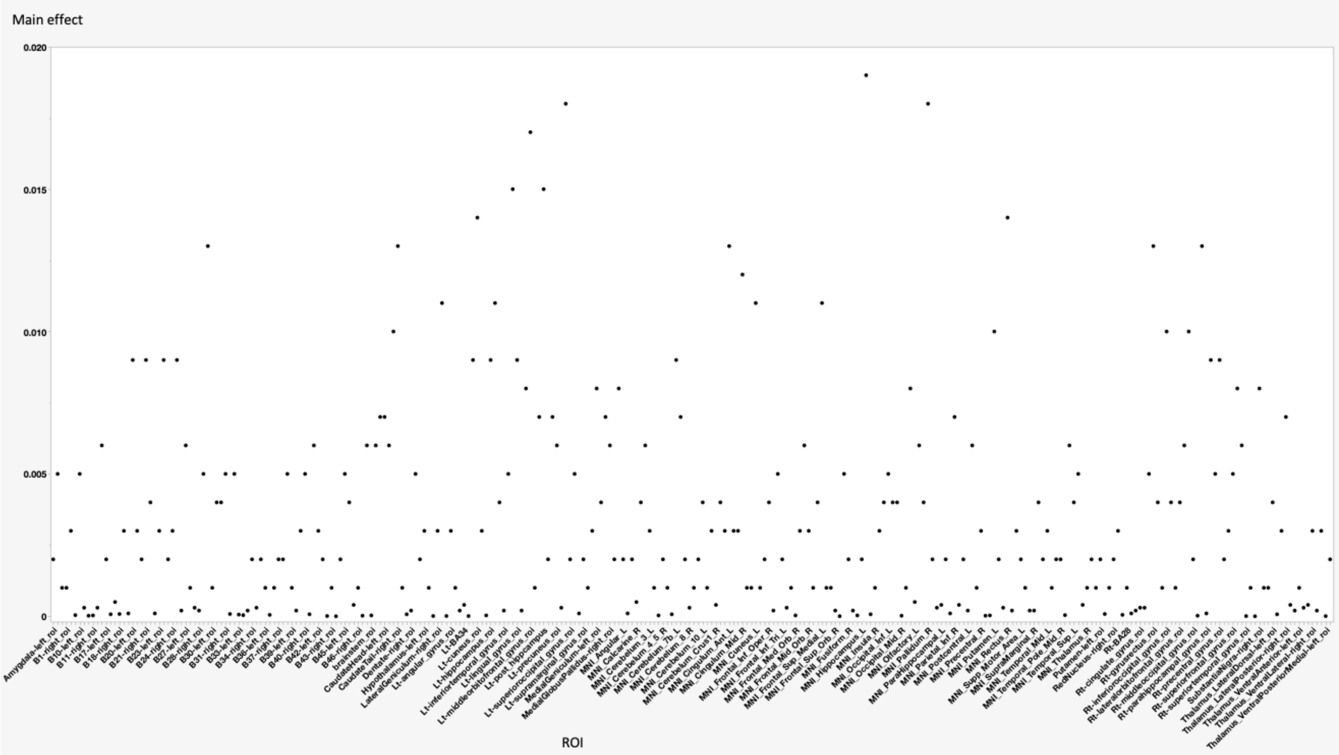

To address the potential impact of multicollinearity among the 290 regions of interest (ROIs), an Elastic Net regression model was employed to evaluate the main effect of each neuroanatomical feature. The plot illustrates the relative contribution (coefficients) of individual ROIs to the predictive model. The Elastic Net approach, which combines L1 and L2 regularization, effectively performs feature selection and handles highly correlated variables, ensuring that the identified main effects are robust and representative. Each dot represents a specific ROI along the x-axis, with its corresponding predictive weight shown on the y-axis, highlighting the key structural markers driving the Schizophrenia-like score (SPS).

Supplementary Figure 3. Consistency of morphometric alterations in schizophrenia across five independent databases.

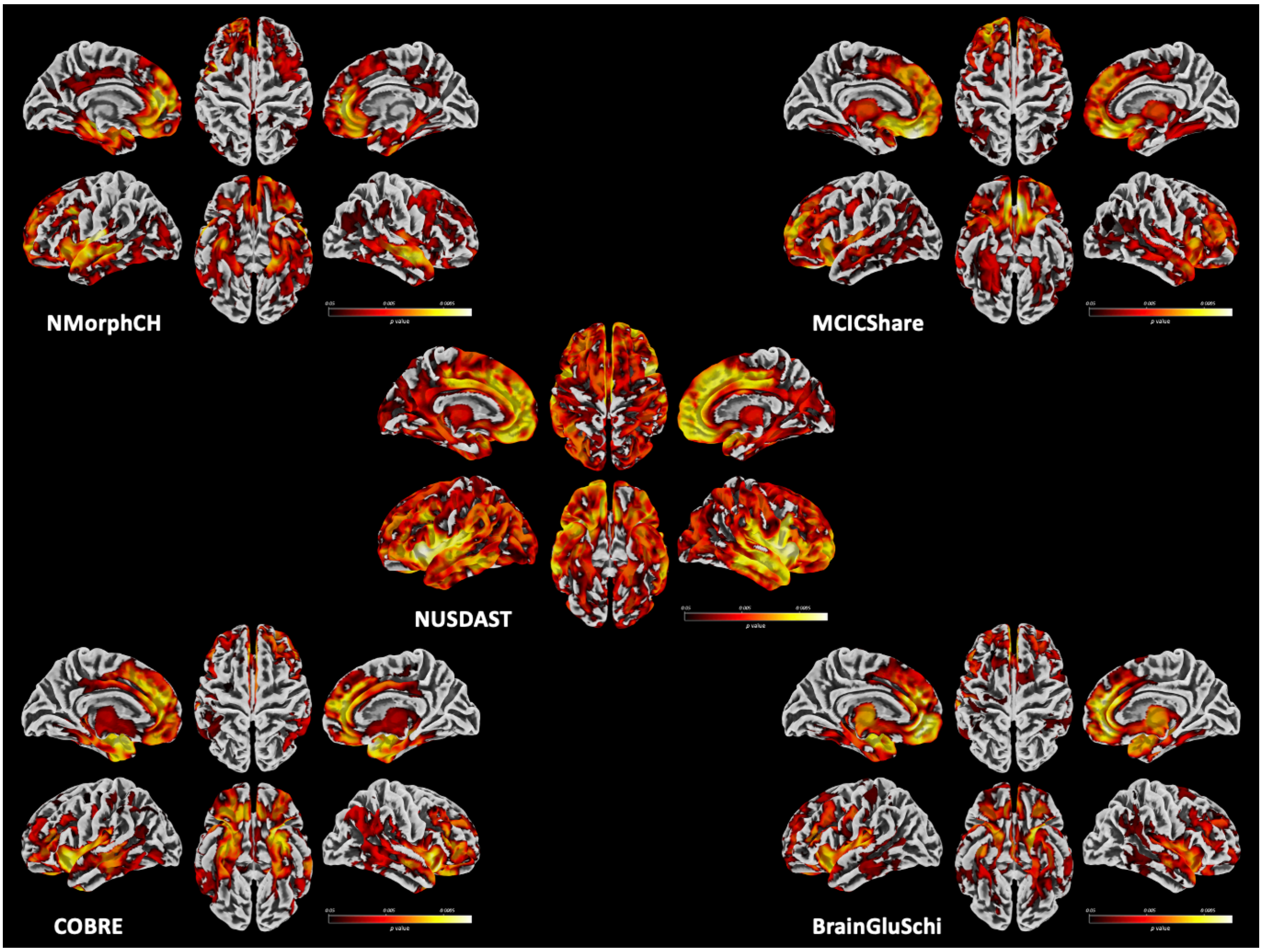

Voxel-based morphometry (VBM) maps, enhanced by Threshold-Free Cluster Enhancement (TFCE), demonstrate that structural brain changes in schizophrenia are highly consistent across different datasets (NMorphPH, MCICShare, NUSDAST, COBRE, and BrainGluSchi). Despite differences in scan sites and protocols, a common pattern of gray matter reduction is observed across all databases, particularly in the prefrontal cortex, superior temporal gyrus, and subcortical structures including the thalamus and striatum. These findings confirm that the identified morphometric signatures are robust and representative of the schizophrenia phenotype, rather than being site-specific artifacts.

Supplementary Figure 4: Scatter plots of the z-values of automated anatomical labeling (AAL) regions of interest (ROI) in the schizophrenia group.

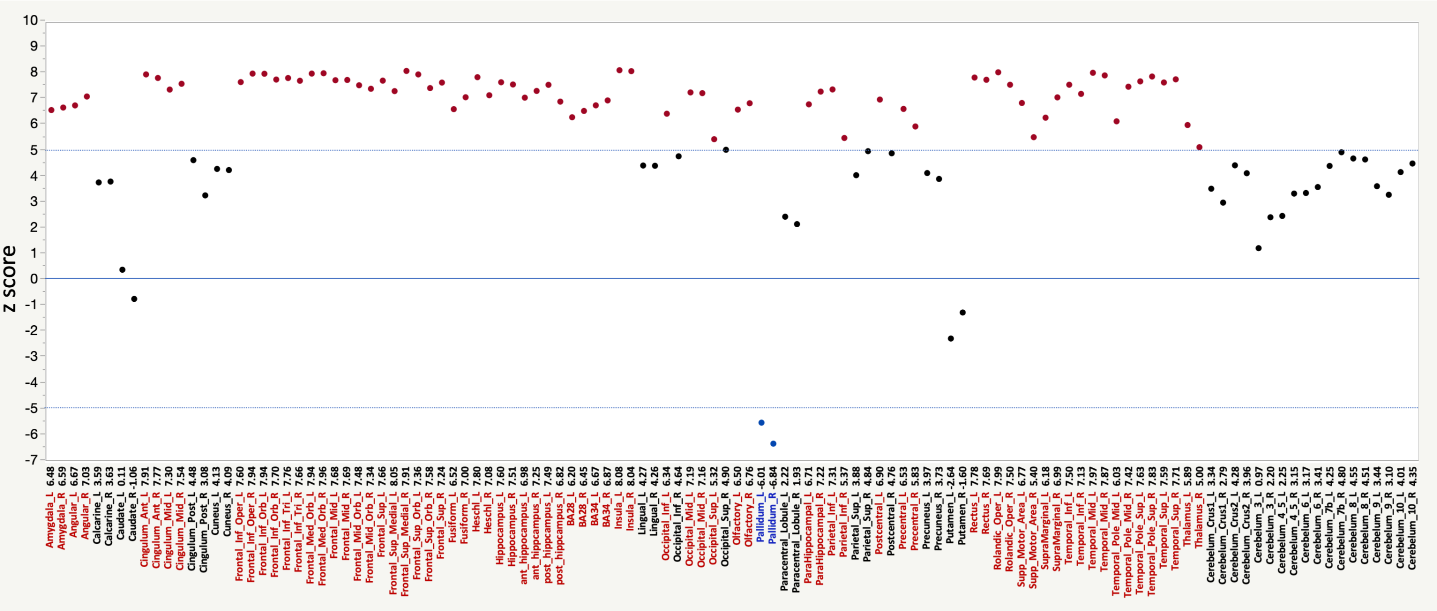

Red letters indicate regions of decreased volume, whereas blue letters indicate regions of increased volume; The threshold level of Z value for family-wise error (FWE) < 0.05 was 4.9.

Supplementary Figure 5: Effectiveness of TIV correction in normalizing sex differences in brain volumes.

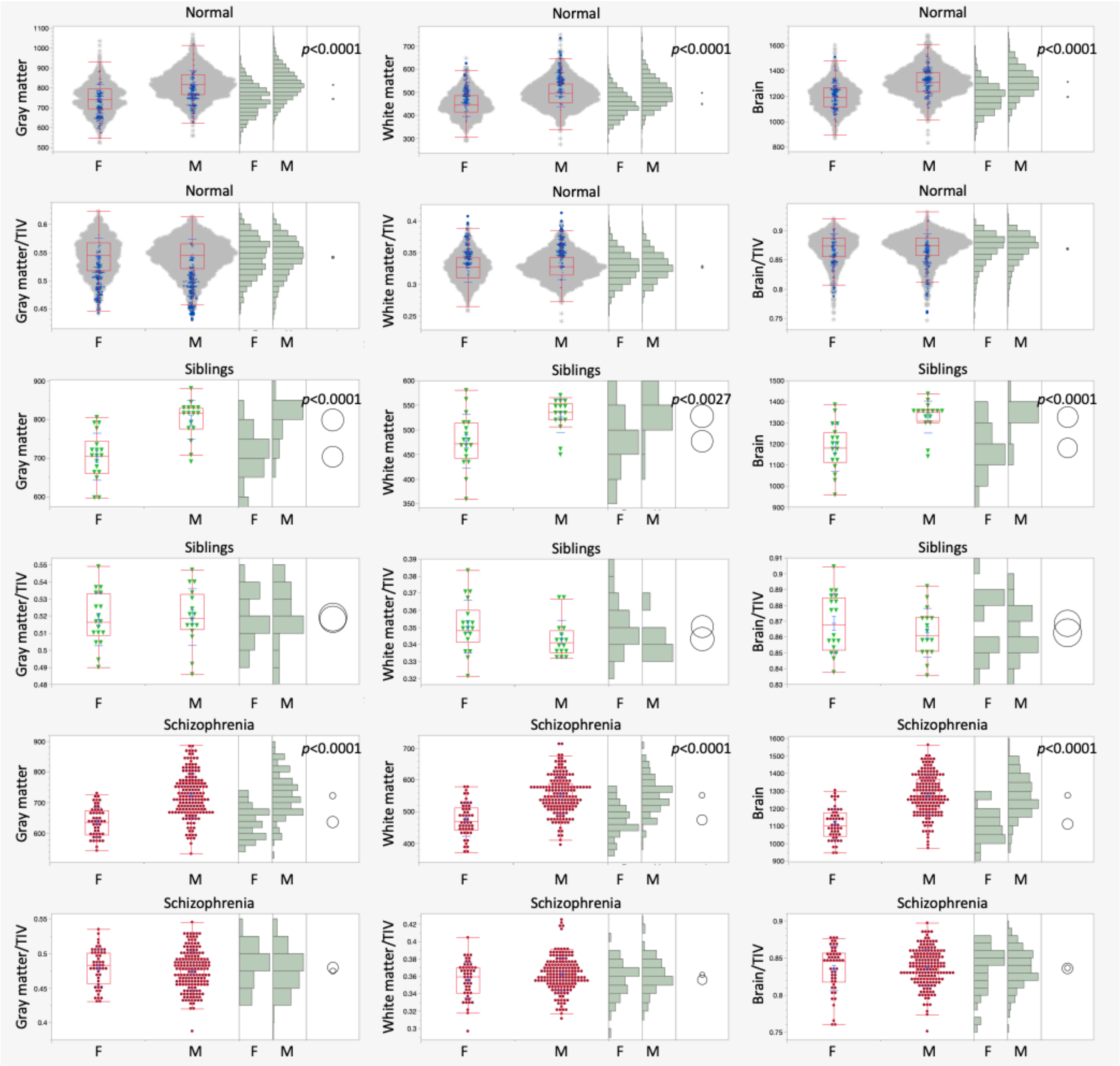

Comparison of brain volumes between females (F) and males (M) in healthy controls (Normal), siblings, and patients with schizophrenia. The upper rows for each category show absolute volumes for gray matter, white matter, and total brain, where significant sex differences were observed (Student's t-test, $p < 0.0001$). The lower rows display the same metrics after correction for total intracranial volume (TIV-corrected ratio). Following TIV correction, the volumetric differences between males and females were no longer statistically significant, demonstrating that TIV correction effectively accounts for inherent sex-related variations in head size.

Supplementary Figure 6：Representative brain MR images and morphometric analysis.

Illustrative case for clinical diagnosis on MRI, Case A

Case A; 37 years old, M

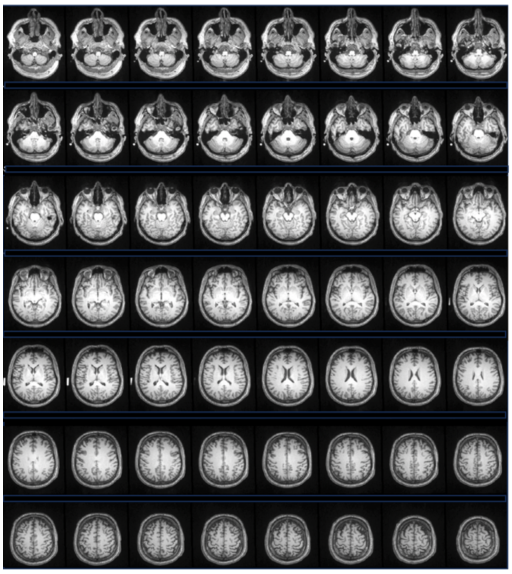

SPS; 0.92

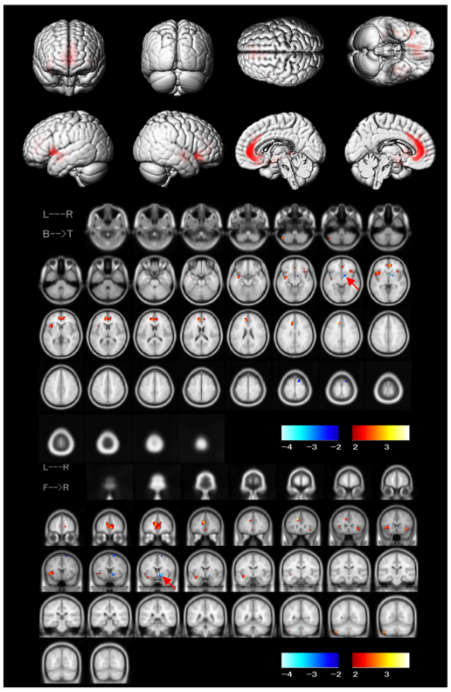

Illustrative case for clinical diagnosis on MRI, Case B

Case B; 22 years old, M

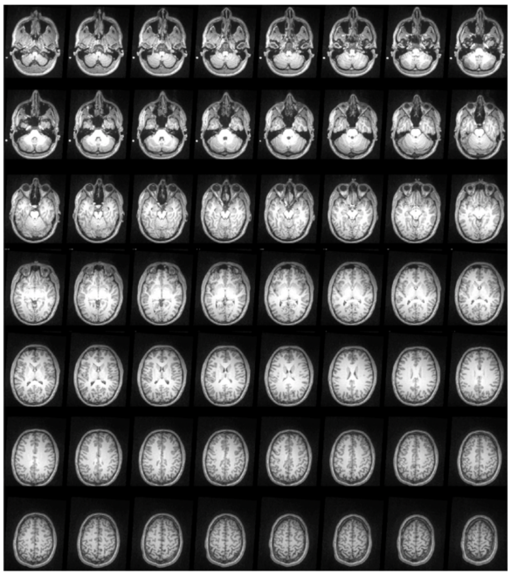

SPS; 0.29

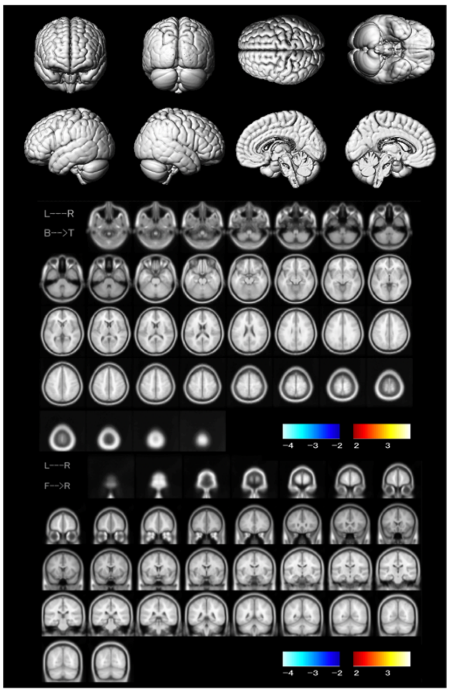

Structural MR images of a 37-year-old male with schizophrenia (Case A) and a 22-year-old healthy male (Case B), both randomly selected from the COBRE database. Voxel-based morphometry (VBM) and machine learning results are displayed to the right of each MRI. Case A exhibits prominent atrophy

in the anterior cingulate and insular cortices, alongside enlargement of the right pallidum (red arrow), whereas Case B shows no detectable structural abnormalities. The machine learning-derived SPS for Case A was 0.92, indicating a high probability of schizophrenia. In contrast, Case B had an SPS of 0.29, consistent with a low likelihood of the disorder.

Supplementary Figure 7: Impact of ComBat-GAM on Effect Size (Cohen's d) across 290 ROIs

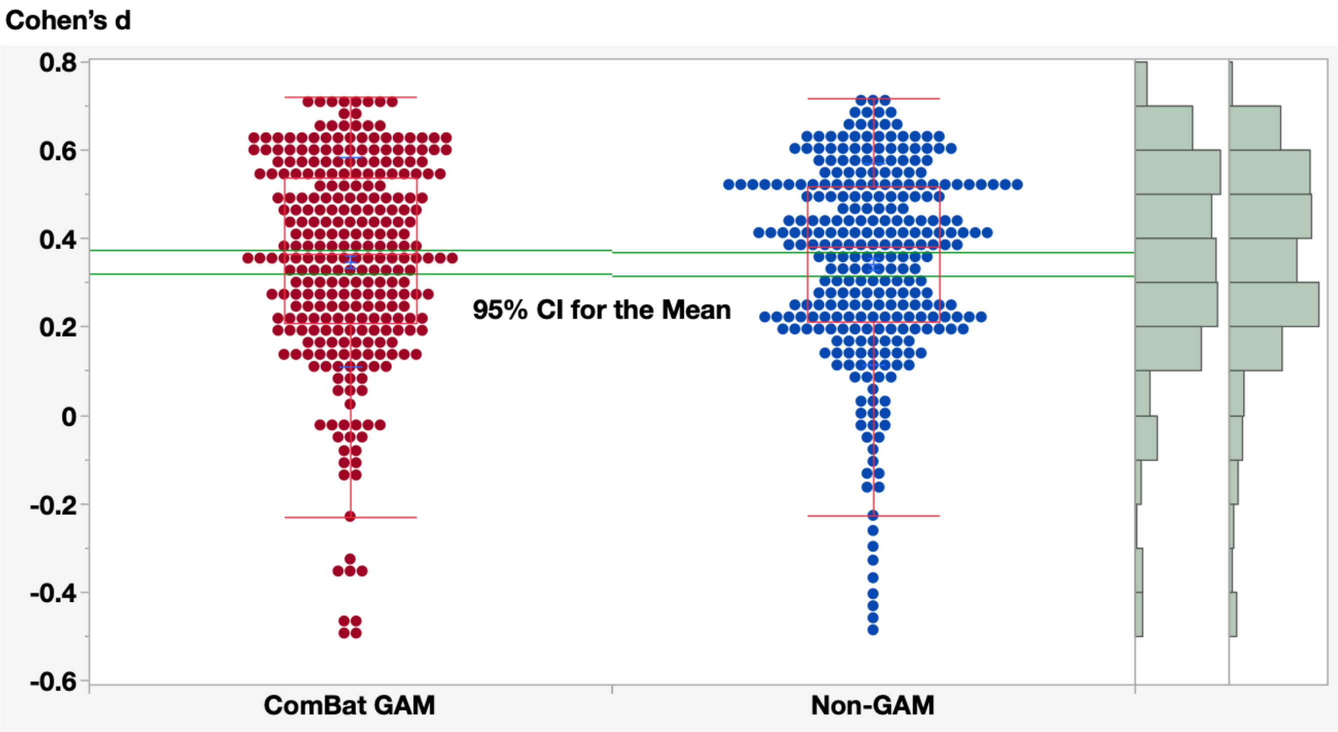

This figure illustrates the distribution of Cohen's d values for 290 regions of interest (ROIs) in the differentiation between healthy controls and patients with schizophrenia, comparing results before and after the application of ComBat-GAM. The red plot represents the "ComBat GAM" group, while the blue plot shows the "Non-GAM" group. The green horizontal lines indicate the 95% confidence interval (CI) for the mean of Cohen's d for each method.

The mean Cohen's d values for the differentiation between healthy controls and patients with schizophrenia were 0.341 for the non-GAM approach and 0.346 after applying ComBat-GAM. While the distribution of effect sizes across the 290 ROIs appears similar between the two methods, ComBat-GAM yielded a slightly higher mean value.

Supplementary Figure 8：Statistical Parametric Mapping (SPM) Report

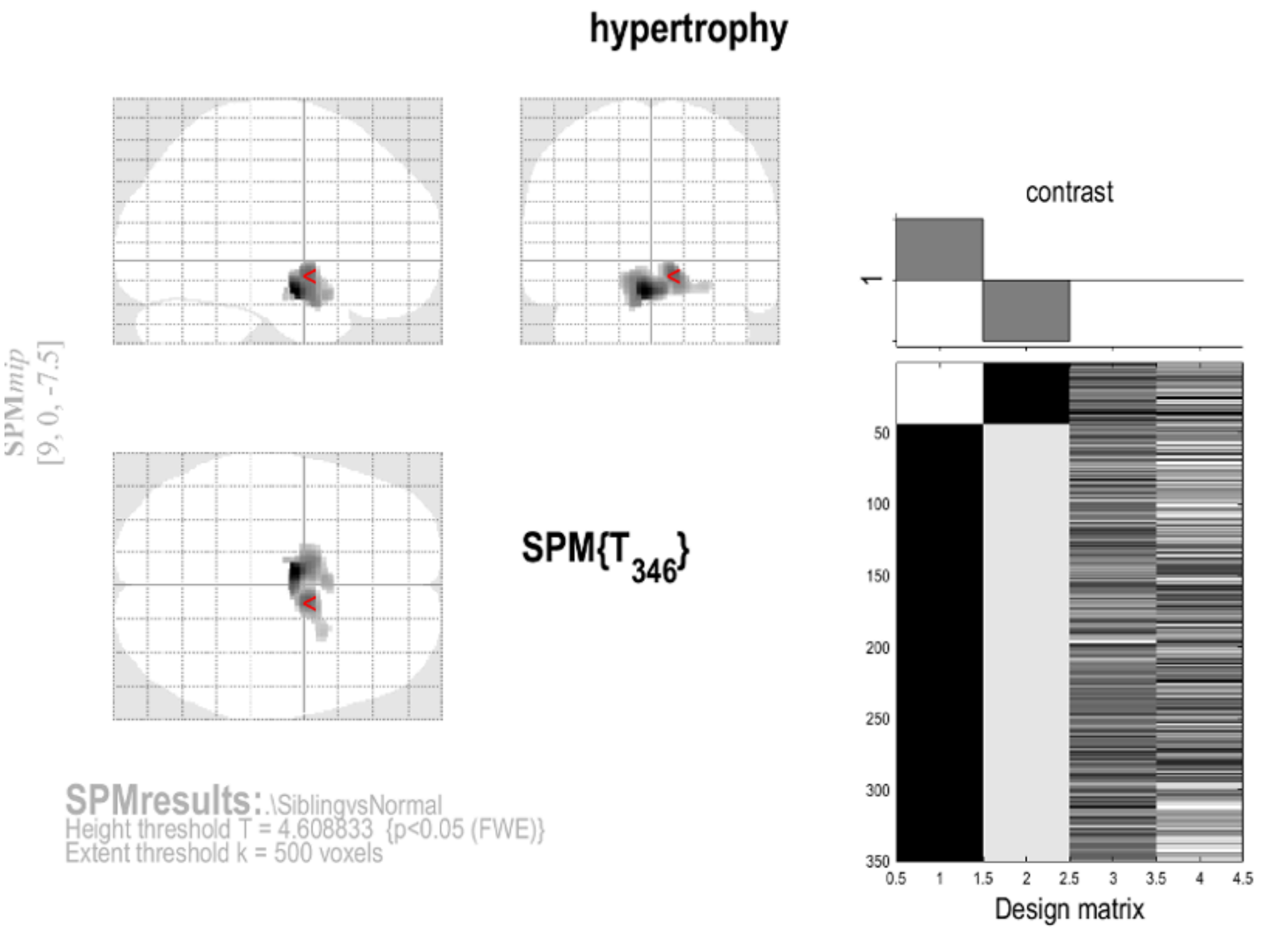

Statistics: ***p-values adjusted for search volume***

cluster-level				peak-level					mm mm mm		
$p_{FWE\text{-}corr}$	$q_{FDR\text{-}corr}$	k_E	p_{uncorr}	$p_{FWE\text{-}corr}$	$q_{FDR\text{-}corr}$	T	(Z_E)	p_{uncorr}			
0.000	0.000	1461	0.000	0.000	0.000	7.00	6.77	0.000	-6	-8	-16
				0.000	0.035	5.84	5.70	0.000	9	0	-8
				0.004	0.224	5.22	5.11	0.000	-2	9	-20

table shows 3 local maxima more than 8.0mm apart

Height threshold: T = 4.61, p = 0.000 (0.050)
Extent threshold: k = 500 voxels, p = 0.001 (0.000)
Expected voxels per cluster, <k> = 33.394
Expected number of clusters, <c> = 0.00
FWEp: 4.609, FDRp: 5.838, FWEc: 1, FDRc: 278

Degrees of freedom = [1.0, 346.0]
FWHM = 15.1 14.8 14.4 mm mm mm; 10.1 9.8 9.6 {voxels}
Volume: 1640412 = 486048 voxels = 459.8 resels
Voxel size: 1.5 1.5 1.5 mm mm mm; (resel = 950.44 voxels)

1. Statistical Thresholding and Theory (Methods/Analysis)

Statistical inference was performed based on the Gaussian Random Field Theory (RFT). To account for the spatial correlation between voxels, the search volume was estimated to be 459.8 resels (resolution elements), with an estimated spatial smoothness (FWHM) of approximately 15 mm (15.1×14.8 ×14.4mm). Multiple comparison correction was applied at the cluster level using Family-Wise Error (FWE) correction to maintain a stringent significance threshold.

2. Results

The two-group comparison revealed a statistically robust difference within a single large cluster (kE =1461 voxels; $p_{FWE-corr} < 0.001$). This cluster significantly exceeded the predefined extent threshold (k=500 voxels), indicating a highly reliable group effect across the reward-related network.
The peak of this cluster was identified in the left midbrain (MNI: −6, −8, −16; T=7.00, Z_E=6.77, $p_{FWE-corr}$< 0.001), corresponding to the ventral tegmental area (VTA). Within the same significant cluster, a distinct local maximum was observed in the right basal forebrain (MNI: 9, 0, −8; T=5.84), which encompasses the nucleus accumbens (NAcc) and adjacent reward-related structures. Additionally, the cluster extended into the medial orbitofrontal cortex (mOFC) and the subgenual region (MNI: −2, 9, −20; T=5.22).

Structural Neuroimaging Analysis (BAAD)

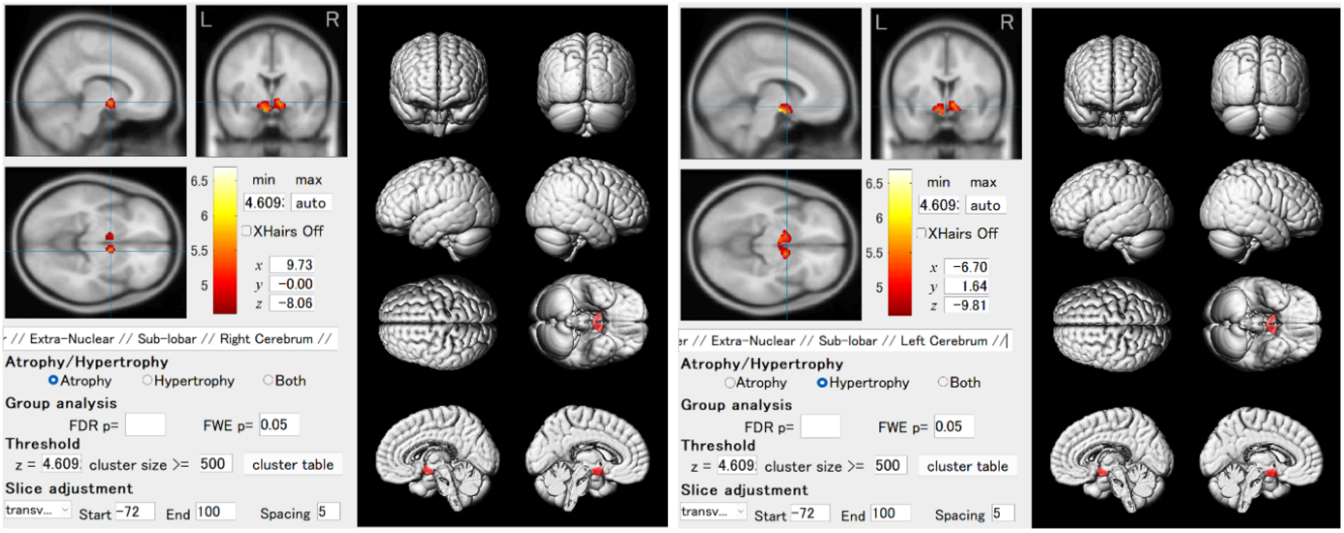

Voxel-based morphometry (VBM) was performed using the BAAD (Brain Anatomical Analysis using Diffeomorphic deformation) software, which implements an automated pipeline based on the Statistical Parametric Mapping (SPM) engine. Statistical inference was based on Gaussian Random Field Theory, with a search volume of 395.7 resels and an estimated spatial smoothness (FWHM) of 15 mm. To identify potential structural markers of resilience, we compared unaffected siblings of patients with schizophrenia to healthy controls.
The two-group comparison revealed a statistically robust cluster of increased gray matter volume (hypertrophy) in the unaffected siblings (k_E=1461 voxels; $p_{FWE-corr}$<0.001). This cluster, which encompasses the core nodes of the mesolimbic reward network, demonstrates significant structural expansion at key anatomical locations.
As illustrated in the provided figures, detailed voxel-based mapping identified two prominent points of hypertrophy within this network (z=4.609; cluster size $\geq$ 500 voxels; p_{FWE}=0.05):

1. Right Nucleus Accumbens (NAcc): In the right cerebrum, a significant cluster was identified in the sub-lobar, extra-nuclear region (MNI: x=9.73, y=−0.00, z=−8.06; z=4.609). The orthogonal slices and 3D surface reconstructions visualize this expansion within the ventral striatum.

2. Left Midbrain (VTA): Similarly, in the left cerebrum, a significant cluster was located in the midbrain/sub-lobar region (MNI: $x=-6.70, y=1.64, z=-9.81;\ z=4.609$), a location consistent with the ventral tegmental area (VTA).

The 3D surface renderings (right panels) further highlight the spatial distribution of this hypertrophy deep within the midbrain and basal ganglia. These bilateral findings of structural expansion in the VTA and NAcc underscore a comprehensive reinforcement of the reward system. Such a neuroanatomical signature in unaffected siblings provides evidence of a potential "resilience phenotype" against the genetic risk for schizophrenia.

Supplementary Tables

Logic for Retaining All 290 ROIs in the Model

1. Integration of Global Morphometric Patterns

Although some individual ROIs did not reach statistical significance ($p<0.05$), the Total Effect values across the 290 regions indicate that biological signals are distributed throughout the brain rather than localized to a few areas. By retaining the full set of ROIs, the model captures the global morphometric pattern of the disease. Excluding variables based solely on p-values or multicollinearity risks losing these subtle but cumulative contributions, which collectively enhance the model's discriminative power.

2. Preservation of Asymmetrical Clinical Information

Brain structures exhibit high inter-hemispheric correlation; however, neurodegenerative diseases often manifest as asymmetrical atrophy. Our results show varying Estimates and Total Effects between left and right homologous regions. We utilized Elastic Net regularization specifically because it is mathematically designed to handle multicollinearity by distributing weights among correlated variables rather than arbitrarily discarding them. This approach allows the model to preserve clinically relevant lateralization that would be lost through traditional variable selection.

3. Empirical Justification via Predictive Performance

Empirical validation demonstrated that reducing the number of ROIs did not improve—and in some cases, diminished—the classification accuracy on the test dataset. This suggests that the inclusion of all 290 ROIs provides a necessary level of granularity for the Support Vector Machine (SVM) to establish a robust decision boundary. The stability of the test-set performance confirms that the model is not merely overfitted to noise, but is instead utilizing the summation of weak signals across the entire brain to achieve optimal generalization.

As clearly shown in the Main Effect plot (Supplementary Figure 2), the contribution to the predictive model is distributed across the entire brain. While we acknowledge that a portion of the 290 ROIs exhibits Main Effects near zero, this is a characteristic result of Elastic Net regularization, which effectively shrinks the coefficients of less contributory or redundant variables to maintain model stability.

However, these 'near-zero' regions should not be dismissed as irrelevant; rather, they form a 'long tail' of minor effects that collectively provides the necessary information for the SVM to achieve high classification accuracy. Empirically, our trials confirmed that removing these regions—even those with minimal individual effects—led to a consistent drop in predictive performance on the test dataset. This suggests that these subtle signals provide an essential baseline for the model to accurately distinguish the complex, distributed pathological patterns of schizophrenia from normal variation. Therefore, we retained the full anatomical set to ensure a comprehensive, data-driven representation of brain-wide structural alterations, avoiding the information loss inherent in manual feature exclusion.

Supplementary Table 1: **Statistical analysis of ROIs related to schizophrenia diagnosis: Evaluation of main effects and multicollinearity using Elastic Net regression.**

ROI	P-value	FDR Logworth	Effect Size	Fractional Rank	Bootstrap Estimates	Non-Zero Percentage	VIF	Estimate	Std Error	Wald ChiSquare	Main Effect
Lt-ant_hippocampus	0.00	14.63	0.25	0.25	0.04	0.34	405.62	0.15	1.79	0.01	0.00
Lt-post_hippcampus	0.00	18.23	0.29	0.14	-0.20	0.41	177.16	-1.18	1.24	0.91	0.02
Rt-ant_hippcampus	0.00	17.26	0.28	0.17	0.00	0.13	372.39	0.03	1.94	0.00	0.00
Rt-post_hippcampus	0.00	15.84	0.27	0.20	0.18	0.57	193.61	0.81	1.65	0.24	0.01
Lt-BA28	0.00	8.54	0.19	0.51	0.00	0.07	3639.97	0.06	4.00	0.00	0.00
Lt-BA34	0.00	10.26	0.21	0.41	0.06	0.37	108490.97	0.14	23.77	0.00	0.00
Rt-BA28	0.00	7.88	0.18	0.54	-0.12	0.69	5472.67	-0.20	4.23	0.00	0.00
Rt-BA34	0.00	10.05	0.21	0.43	0.00	0.04	103608.80	0.06	23.10	0.00	0.00
MNI_Amygdala_L	0.00	10.56	0.21	0.39	0.15	0.69	615.89	0.24	1.80	0.02	0.00
MNI_Amygdala_R	0.00	9.99	0.21	0.44	0.08	0.34	93.34	0.60	0.66	0.84	0.01
MNI_Angular_L	0.00	7.60	0.18	0.55	-0.14	0.72	9.91	-0.36	0.29	1.51	0.00
MNI_Angular_R	0.00	11.05	0.22	0.37	0.15	0.91	15.54	-0.08	0.39	0.04	0.00
MNI_Calcarine_L	0.02	1.63	0.07	0.89	-0.01	0.07	133.01	-0.31	1.17	0.07	0.00
MNI_Calcarine_R	0.00	2.73	0.10	0.82	0.13	0.64	71.85	0.14	0.74	0.03	0.00
MNI_Caudate_L	0.36	0.43	0.03	0.96	0.00	0.05	825.82	0.40	2.53	0.03	0.00
MNI_Caudate_R	0.05	1.28	0.06	0.91	0.08	0.20	294.62	0.65	1.47	0.20	0.01
MNI_Cingulum_Ant_L	0.00	21.52	0.32	0.04	0.31	0.90	114.04	1.07	0.92	1.35	0.01
MNI_Cingulum_Ant_R	0.00	19.32	0.30	0.10	-0.08	0.33	69.09	-0.50	0.73	0.47	0.00
MNI_Cingulum_Mid_L	0.00	12.90	0.24	0.32	-0.09	0.43	138.38	-0.39	0.98	0.16	0.00
MNI_Cingulum_Mid_R	0.00	14.96	0.26	0.23	0.18	0.57	90.18	0.94	0.78	1.45	0.01
MNI_Cingulum_Post_L	0.00	4.70	0.14	0.64	-0.02	0.43	30.26	-0.29	0.55	0.28	0.00
MNI_Cingulum_Post_R	0.00	2.82	0.10	0.80	0.02	0.39	21.93	0.29	0.45	0.42	0.00
MNI_Cuneus_L	0.00	2.80	0.10	0.81	-0.23	0.76	43.28	-1.08	0.57	3.54	0.01
MNI_Cuneus_R	0.00	2.43	0.09	0.83	-0.05	0.41	43.43	-0.23	0.52	0.19	0.00
MNI_Frontal_Inf_Oper_L	0.00	15.14	0.26	0.22	0.11	0.67	39.07	0.30	0.52	0.33	0.00
MNI_Frontal_Inf_Oper_R	0.00	16.98	0.28	0.17	0.24	0.90	22.55	0.40	0.45	0.78	0.00
MNI_Frontal_Inf_Orb_L	0.00	19.22	0.29	0.12	-0.01	0.13	104.27	0.13	0.91	0.02	0.00
MNI_Frontal_Inf_Orb_R	0.00	16.90	0.27	0.17	-0.06	0.22	109.53	-0.68	0.87	0.61	0.01
MNI_Frontal_Inf_Tri_L	0.00	18.07	0.28	0.15	0.07	0.48	44.54	0.38	0.54	0.51	0.00
MNI_Frontal_Inf_Tri_R	0.00	14.36	0.25	0.27	-0.06	0.36	50.70	-0.15	0.63	0.05	0.00
MNI_Frontal_Med_Orb_L	0.00	21.25	0.31	0.05	0.09	0.61	20.80	0.31	0.36	0.74	0.00
MNI_Frontal_Med_Orb_R	0.00	23.16	0.33	0.02	0.05	0.58	19.38	-0.03	0.37	0.01	0.00
MNI_Frontal_Mid_L	0.00	14.99	0.26	0.23	0.09	0.63	56.45	0.37	0.56	0.43	0.00
MNI_Frontal_Mid_Orb_L	0.00	15.46	0.26	0.20	0.10	0.50	31.17	0.59	0.46	1.63	0.01
MNI_Frontal_Mid_Orb_R	0.00	11.70	0.23	0.35	-0.17	0.86	36.02	-0.37	0.47	0.61	0.00
MNI_Frontal_Mid_R	0.00	15.29	0.26	0.22	0.04	0.38	73.23	0.20	0.73	0.07	0.00
MNI_Frontal_Sup_L	0.00	14.65	0.25	0.25	0.08	0.39	44.00	0.48	0.55	0.77	0.00
MNI_Frontal_Sup_Medial_L	0.00	20.55	0.31	0.06	0.44	0.98	27.01	0.92	0.37	6.33	0.01
MNI_Frontal_Sup_Medial_R	0.00	18.39	0.29	0.14	0.13	0.75	24.06	0.21	0.46	0.20	0.00
MNI_Frontal_Sup_Orb_L	0.00	12.93	0.24	0.31	-0.02	0.44	29.17	0.19	0.36	0.26	0.00
MNI_Frontal_Sup_Orb_R	0.00	13.34	0.24	0.30	-0.10	0.70	35.16	-0.08	0.39	0.04	0.00
MNI_Frontal_Sup_R	0.00	10.52	0.21	0.40	-0.19	0.88	59.38	0.00	0.62	0.00	0.00
MNI_Fusiform_L	0.00	9.53	0.20	0.46	-0.28	0.78	58.86	-0.49	0.67	0.54	0.01
MNI_Fusiform_R	0.00	11.16	0.22	0.37	0.19	0.70	75.39	0.33	0.73	0.20	0.00
MNI_Heschl_L	0.00	19.65	0.30	0.09	0.03	0.33	30.31	0.11	0.49	0.05	0.00
MNI_Heschl_R	0.00	14.32	0.25	0.27	-0.01	0.25	34.86	-0.04	0.58	0.00	0.00
MNI_Hippocampus_L	0.00	19.72	0.30	0.09	0.00	0.03	581.06	0.22	2.28	0.01	0.00
MNI_Hippocampus_R	0.00	20.91	0.31	0.05	0.71	0.99	700.30	1.40	2.89	0.24	0.02
MNI_Insula_L	0.00	22.78	0.32	0.03	0.02	0.18	415.99	-0.06	1.74	0.00	0.00
MNI_Insula_R	0.00	23.61	0.33	0.01	0.03	0.10	888.93	0.27	2.66	0.01	0.00
MNI_Lingual_L	0.00	3.06	0.11	0.77	-0.28	0.82	50.16	-0.53	0.72	0.55	0.00
MNI_Lingual_R	0.00	3.71	0.12	0.72	0.01	0.14	67.83	0.48	0.72	0.44	0.00
MNI_Occipital_Inf_L	0.00	8.61	0.19	0.50	-0.10	0.67	17.70	-0.52	0.38	1.88	0.01
MNI_Occipital_Inf_R	0.00	4.67	0.14	0.65	0.27	0.92	13.24	0.53	0.37	2.06	0.00
MNI_Occipital_Mid_L	0.00	10.83	0.22	0.38	0.01	0.42	35.59	-0.52	0.67	0.62	0.00
MNI_Occipital_Mid_R	0.00	10.33	0.21	0.41	0.12	0.72	33.20	0.01	0.50	0.00	0.00
MNI_Occipital_Sup_L	0.00	4.68	0.14	0.64	0.04	0.40	23.89	-0.22	0.50	0.19	0.00
MNI_Occipital_Sup_R	0.00	3.56	0.12	0.72	-0.20	0.74	27.04	-0.76	0.44	2.99	0.01
MNI_Olfactory_L	0.00	7.76	0.18	0.55	-0.09	0.54	109.04	-0.18	0.84	0.05	0.00
MNI_Olfactory_R	0.00	8.93	0.19	0.49	-0.13	0.42	102.72	-0.69	0.89	0.60	0.01
MNI_Pallidum_L	0.00	10.01	0.21	0.43	-0.04	0.15	160.08	-0.44	0.99	0.20	0.00
MNI_Pallidum_R	0.00	12.84	0.24	0.33	-0.57	0.94	123.65	-1.42	0.80	3.20	0.02
MNI_Paracentral_Lobule_L	0.72	0.13	0.01	0.98	-0.12	0.85	16.36	-0.35	0.33	1.15	0.00
MNI_Paracentral_Lobule_R	0.80	0.09	0.01	0.99	0.02	0.45	12.53	0.12	0.30	0.16	0.00
MNI_ParaHippocampal_L	0.00	10.31	0.21	0.41	0.00	0.11	180.10	0.10	1.03	0.01	0.00
MNI_ParaHippocampal_R	0.00	13.15	0.24	0.31	-0.05	0.24	118.09	-0.28	0.71	0.15	0.00
MNI_Parietal_Inf_L	0.00	10.42	0.21	0.40	0.04	0.44	34.19	-0.09	0.49	0.04	0.00

MNI_Parietal_Inf_R	0.00	4.24	0.13	0.68	-0.36	0.97	30.39	-0.97	0.50	3.73	0.01
MNI_Parietal_Sup_L	0.00	2.60	0.10	0.83	-0.07	0.76	11.63	-0.15	0.32	0.23	0.00
MNI_Parietal_Sup_R	0.00	2.96	0.10	0.79	-0.04	0.55	10.07	-0.35	0.30	1.38	0.00
MNI_Postcentral_L	0.00	8.47	0.19	0.52	0.07	0.45	57.33	-0.13	0.67	0.04	0.00
MNI_Postcentral_R	0.00	2.34	0.09	0.84	-0.25	0.80	58.26	-0.83	0.67	1.53	0.01
MNI_Precentral_L	0.00	5.43	0.15	0.62	-0.03	0.46	45.65	-0.20	0.64	0.10	0.00
MNI_Precentral_R	0.00	3.54	0.12	0.73	-0.07	0.56	45.28	-0.34	0.54	0.39	0.00
MNI_Precuneus_L	0.00	2.87	0.10	0.80	-0.03	0.31	151.52	0.03	1.09	0.00	0.00
MNI_Precuneus_R	0.00	3.02	0.11	0.78	-0.10	0.59	98.28	0.06	0.87	0.00	0.00
MNI_Putamen_L	0.00	3.39	0.11	0.74	0.22	0.60	673.07	0.91	2.12	0.18	0.01
MNI_Putamen_R	0.01	1.96	0.08	0.87	0.03	0.15	486.56	0.32	1.91	0.03	0.00
MNI_Rectus_L	0.00	18.59	0.29	0.13	0.09	0.46	176.29	0.15	1.06	0.02	0.00
MNI_Rectus_R	0.00	16.36	0.27	0.18	0.30	0.62	128.25	1.34	0.92	2.12	0.01
MNI_Rolandic_Oper_L	0.00	22.26	0.32	0.03	0.01	0.29	36.71	0.09	0.49	0.04	0.00
MNI_Rolandic_Oper_R	0.00	14.99	0.26	0.23	-0.22	0.76	46.69	-0.45	0.54	0.70	0.00
MNI_Supp_Motor_Area_L	0.00	6.24	0.16	0.60	0.00	0.52	18.94	0.30	0.36	0.71	0.00
MNI_Supp_Motor_Area_R	0.00	3.40	0.11	0.73	-0.09	0.67	21.65	-0.27	0.38	0.50	0.00
MNI_SupraMarginal_L	0.00	8.47	0.19	0.52	-0.04	0.45	14.24	-0.09	0.32	0.09	0.00
MNI_SupraMarginal_R	0.00	9.35	0.20	0.47	0.03	0.46	34.06	-0.09	0.46	0.04	0.00
MNI_Temporal_Inf_L	0.00	14.71	0.26	0.24	-0.01	0.12	133.35	-0.42	0.90	0.22	0.00
MNI_Temporal_Inf_R	0.00	10.56	0.21	0.39	-0.20	0.77	160.57	-0.34	1.00	0.12	0.00
MNI_Temporal_Mid_L	0.00	19.27	0.30	0.10	0.01	0.16	105.47	0.42	0.81	0.27	0.00
MNI_Temporal_Mid_R	0.00	18.39	0.29	0.13	0.02	0.09	170.32	0.27	1.02	0.07	0.00
MNI_Temporal_Pole_Mid_L	0.00	6.34	0.16	0.59	-0.12	0.71	64.74	-0.33	0.65	0.26	0.00
MNI_Temporal_Pole_Mid_R	0.00	12.45	0.23	0.33	0.16	0.81	44.97	0.31	0.45	0.47	0.00
MNI_Temporal_Pole_Sup_L	0.00	14.63	0.25	0.26	0.02	0.26	149.58	-0.03	0.86	0.00	0.00
MNI_Temporal_Pole_Sup_R	0.00	16.69	0.27	0.18	-0.03	0.32	78.76	-0.58	0.68	0.73	0.01
MNI_Temporal_Sup_L	0.00	15.41	0.26	0.21	-0.19	0.70	71.91	-0.49	0.69	0.50	0.00
MNI_Temporal_Sup_R	0.00	18.37	0.29	0.14	0.16	0.66	77.69	0.55	0.71	0.61	0.01
MNI_Thalamus_L	0.00	4.66	0.14	0.65	0.00	0.00	2305.30	-0.13	3.75	0.00	0.00
MNI_Thalamus_R	0.00	3.11	0.11	0.76	0.00	0.00	4770.02	-0.17	5.37	0.00	0.00
MNI_Cerebelum_Crus1_L	0.00	2.38	0.09	0.84	0.12	0.61	24.59	0.41	0.43	0.88	0.00
MNI_Cerebelum_Crus1_R	0.02	1.66	0.07	0.89	-0.06	0.44	20.61	-0.15	0.43	0.12	0.00
MNI_Cerebelum_Crus2_L	0.00	3.14	0.11	0.75	0.23	0.80	22.57	0.65	0.48	1.84	0.00
MNI_Cerebelum_Crus2_R	0.00	2.65	0.10	0.82	-0.08	0.29	29.09	-0.44	0.53	0.68	0.00
MNI_Cerebelum_3_L	0.19	0.70	0.04	0.96	-0.25	0.88	12.38	-0.48	0.38	1.57	0.00
MNI_Cerebelum_3_R	0.02	1.62	0.07	0.90	-0.12	0.62	16.71	-0.24	0.46	0.26	0.00
MNI_Cerebelum_4_5_L	0.04	1.34	0.06	0.91	-0.03	0.35	33.63	0.03	0.55	0.00	0.00
MNI_Cerebelum_4_5_R	0.00	2.73	0.10	0.82	0.04	0.29	37.10	0.30	0.56	0.27	0.00
MNI_Cerebelum_6_L	0.00	2.76	0.10	0.81	0.02	0.26	30.85	0.20	0.54	0.14	0.00
MNI_Cerebelum_6_R	0.00	3.01	0.11	0.78	0.01	0.24	36.87	-0.07	0.50	0.02	0.00
MNI_Cerebelum_7b_L	0.00	2.81	0.10	0.81	-0.40	0.86	19.29	-1.02	0.36	7.93	0.01
MNI_Cerebelum_7b_R	0.00	3.83	0.12	0.71	0.31	0.91	17.62	0.80	0.39	4.20	0.01
MNI_Cerebelum_8_L	0.00	3.10	0.11	0.76	0.10	0.51	23.53	0.38	0.41	0.84	0.00
MNI_Cerebelum_8_R	0.00	3.92	0.12	0.70	0.10	0.63	24.85	0.14	0.46	0.10	0.00
MNI_Cerebelum_9_L	0.01	2.02	0.08	0.87	0.04	0.42	21.09	0.21	0.39	0.30	0.00
MNI_Cerebelum_9_R	0.02	1.69	0.07	0.88	-0.04	0.41	20.53	-0.35	0.41	0.73	0.00
MNI_Cerebelum_10_L	0.00	3.86	0.12	0.70	0.29	0.97	3.85	0.49	0.18	7.41	0.00
MNI_Cerebelum_10_R	0.00	4.23	0.13	0.68	0.22	0.96	3.72	0.20	0.16	1.51	0.00
Amygdala-left	0.00	9.97	0.21	0.44	0.02	0.14	584.68	-0.24	1.73	0.02	0.00
Amygdala-right	0.00	9.18	0.20	0.48	-0.05	0.20	129.84	-0.48	0.76	0.40	0.01
B1-left	0.00	5.49	0.15	0.62	0.36	1.00	9.74	0.32	0.31	1.06	0.00
B1-right	0.00	3.08	0.11	0.76	0.10	0.65	9.29	0.24	0.26	0.86	0.00
B10-left	0.00	20.76	0.31	0.06	0.22	0.86	24.69	0.49	0.41	1.46	0.01
B10-right	0.00	20.59	0.31	0.06	0.00	0.35	28.55	0.09	0.42	0.04	0.00
B11-left	0.00	14.44	0.25	0.26	-0.02	0.23	134.08	0.02	0.80	0.00	0.00
B11-right	0.00	14.40	0.25	0.26	-0.02	0.17	139.98	0.03	0.80	0.00	0.00
B13-left	0.00	24.67	0.34	0.01	-0.01	0.06	169.75	-0.12	1.04	0.01	0.00
B13-right	0.00	21.32	0.31	0.04	-0.14	0.41	189.02	-0.73	1.29	0.32	0.01
B17-left	0.14	0.84	0.05	0.95	0.15	0.77	29.05	0.31	0.49	0.40	0.00
B17-right	0.11	0.95	0.05	0.94	0.01	0.41	26.71	-0.06	0.47	0.02	0.00
B18-left	0.01	2.22	0.09	0.85	-0.03	0.29	74.15	-0.20	0.75	0.07	0.00
B18-right	0.00	2.27	0.09	0.85	-0.05	0.28	59.64	-0.04	0.78	0.00	0.00
B19-left	0.00	10.17	0.21	0.42	0.06	0.34	43.80	0.40	0.60	0.43	0.00
B19-right	0.00	6.68	0.17	0.58	-0.01	0.19	48.65	-0.10	0.56	0.03	0.00
B2-left	0.00	6.12	0.16	0.60	-0.13	0.59	15.09	-0.42	0.34	1.56	0.00
B2-right	0.00	4.29	0.13	0.68	-0.02	0.36	17.22	0.04	0.38	0.01	0.00
B20-left	0.00	14.32	0.25	0.27	0.30	0.95	83.46	0.76	0.86	0.78	0.01
B20-right	0.00	12.39	0.23	0.34	0.07	0.34	74.76	0.42	0.68	0.38	0.00

B21-left	0.00	15.37	0.26	0.21	-0.07	0.51	47.45	-0.26	0.47	0.32	0.00
B21-right	0.00	20.50	0.31	0.07	0.40	0.91	59.59	0.89	0.61	2.17	0.01
B22-left	0.00	16.32	0.27	0.19	-0.07	0.46	45.56	-0.49	0.49	1.03	0.00
B22-right	0.00	17.72	0.28	0.15	-0.04	0.29	60.04	-0.06	0.70	0.01	0.00
B23-left	0.00	5.79	0.15	0.62	-0.07	0.28	27.26	-0.48	0.49	0.95	0.00
B23-right	0.00	5.89	0.16	0.61	0.34	0.82	30.11	0.98	0.55	3.22	0.01
B24-left	0.00	14.94	0.26	0.24	0.01	0.25	51.08	0.38	0.60	0.39	0.00
B24-right	0.00	12.79	0.24	0.33	-0.05	0.32	33.31	-0.42	0.47	0.78	0.00
B25-left	0.00	12.14	0.23	0.34	0.10	0.18	168.14	0.96	1.10	0.77	0.01
B25-right	0.00	10.87	0.22	0.37	-0.04	0.21	147.19	-0.14	1.01	0.02	0.00
B27-left	0.00	11.37	0.22	0.36	0.32	0.99	19.09	0.58	0.33	3.12	0.01
B27-right	0.00	9.36	0.20	0.47	0.05	0.50	14.36	-0.17	0.34	0.25	0.00
B28-left	0.00	8.04	0.18	0.53	-0.03	0.28	3843.08	-0.09	4.13	0.00	0.00
B28-right	0.00	8.40	0.19	0.53	0.00	0.02	5890.54	-0.06	4.40	0.00	0.00
B29-left	0.10	0.98	0.05	0.93	0.12	0.63	12.87	0.55	0.33	2.81	0.01
B29-right	0.08	1.06	0.05	0.93	-0.53	1.00	18.91	-1.02	0.43	5.58	0.01
B30-left	0.00	3.56	0.12	0.73	0.17	0.75	33.88	0.21	0.62	0.11	0.00
B30-right	0.00	2.87	0.10	0.80	0.09	0.36	60.54	0.49	0.83	0.34	0.00
B31-left	0.00	9.01	0.20	0.49	0.09	0.41	60.12	0.54	0.67	0.65	0.00
B31-right	0.00	7.03	0.17	0.57	-0.15	0.49	46.91	-0.63	0.62	1.01	0.01
B32-left	0.00	21.40	0.31	0.04	0.02	0.19	88.06	-0.04	0.79	0.00	0.00
B32-right	0.00	20.04	0.30	0.08	0.08	0.40	89.87	0.56	0.84	0.44	0.01
B33-left	0.00	5.93	0.16	0.60	-0.03	0.54	8.82	-0.04	0.31	0.01	0.00
B33-right	0.00	6.29	0.16	0.59	-0.02	0.45	6.21	-0.04	0.26	0.03	0.00
B34-left	0.00	10.17	0.21	0.42	0.00	0.04	107721.86	0.08	23.68	0.00	0.00
B34-right	0.00	10.14	0.21	0.42	0.04	0.17	102177.67	0.26	23.08	0.00	0.00
B35-left	0.00	7.44	0.18	0.56	0.06	0.35	41.60	0.10	0.48	0.05	0.00
B35-right	0.00	9.53	0.20	0.45	0.07	0.47	30.13	0.38	0.47	0.65	0.00
B36-left	0.00	9.14	0.20	0.48	-0.13	0.68	39.41	-0.24	0.51	0.21	0.00
B36-right	0.00	9.37	0.20	0.46	-0.02	0.24	28.28	-0.03	0.41	0.00	0.00
B37-left	0.00	13.17	0.24	0.30	0.02	0.35	36.89	0.23	0.55	0.18	0.00
B37-right	0.00	7.81	0.18	0.54	0.01	0.35	32.60	0.30	0.44	0.47	0.00
B38-left	0.00	11.32	0.22	0.36	0.00	0.11	245.20	0.29	1.17	0.06	0.00
B38-right	0.00	16.27	0.27	0.19	0.04	0.28	202.16	0.51	1.10	0.21	0.01
B39-left	0.00	9.86	0.21	0.44	0.12	0.61	26.50	0.28	0.50	0.30	0.00
B39-right	0.00	6.91	0.17	0.57	-0.13	0.78	15.64	-0.12	0.31	0.17	0.00
B40-left	0.00	10.52	0.21	0.40	0.19	0.73	60.12	0.37	0.59	0.40	0.00
B40-right	0.00	8.48	0.19	0.51	0.10	0.33	88.28	0.60	0.74	0.67	0.01
B41-left	0.00	9.70	0.20	0.45	-0.01	0.31	24.37	-0.06	0.46	0.01	0.00
B41-right	0.00	15.53	0.26	0.20	0.16	0.75	19.47	0.47	0.41	1.32	0.01
B42-left	0.00	8.54	0.19	0.50	0.10	0.60	10.86	0.36	0.26	1.90	0.00
B42-right	0.00	7.60	0.18	0.56	-0.06	0.48	11.91	-0.34	0.27	1.55	0.00
B43-left	0.00	8.67	0.19	0.50	0.13	0.84	9.90	0.02	0.31	0.00	0.00
B43-right	0.00	4.51	0.13	0.66	-0.06	0.69	13.44	0.22	0.35	0.39	0.00
B44-left	0.00	10.74	0.22	0.38	-0.05	0.36	25.16	0.00	0.45	0.00	0.00
B44-right	0.00	14.22	0.25	0.28	0.21	0.85	15.43	0.32	0.34	0.90	0.00
B45-left	0.00	6.30	0.16	0.59	-0.39	0.99	14.77	-0.68	0.36	3.60	0.01
B45-right	0.00	7.26	0.17	0.57	-0.19	0.87	16.62	-0.50	0.38	1.72	0.00
B46-left	0.00	8.51	0.19	0.51	0.06	0.48	13.63	0.15	0.32	0.21	0.00
B46-right	0.00	9.38	0.20	0.46	0.08	0.65	12.64	0.28	0.31	0.85	0.00
B47-left	0.00	20.20	0.30	0.07	0.05	0.22	96.36	-0.02	0.73	0.00	0.00
B47-right	0.00	19.72	0.30	0.09	0.17	0.59	122.15	0.68	0.82	0.69	0.01
Caudate-left	0.06	1.20	0.06	0.92	-0.14	0.68	157.77	-0.52	1.05	0.24	0.01
Caudate-right	0.01	2.01	0.08	0.87	-0.33	0.91	81.94	-0.64	0.78	0.68	0.01
CaudateHead-left	0.92	0.04	0.00	1.00	-0.16	0.44	78.73	-0.82	0.81	1.02	0.01
CaudateHead-right	0.89	0.05	0.00	1.00	0.30	0.90	32.62	0.69	0.54	1.62	0.01
CaudateTail-left	0.00	12.84	0.24	0.32	0.42	1.00	19.13	0.94	0.44	4.63	0.01
CaudateTail-right	0.00	9.07	0.20	0.48	-0.30	0.72	17.89	-1.13	0.46	6.03	0.01
Dentate-left	0.07	1.12	0.06	0.92	-0.01	0.46	6.71	-0.06	0.23	0.07	0.00
Dentate-right	0.02	1.68	0.07	0.89	0.02	0.53	6.58	0.09	0.24	0.13	0.00
Hippocampus-left	0.00	19.25	0.29	0.11	0.08	0.34	148.65	0.51	1.06	0.23	0.01
Hippocampus-right	0.00	17.50	0.28	0.16	-0.01	0.07	121.51	-0.26	1.07	0.06	0.00
Hypothalamus-left	0.16	0.77	0.04	0.95	-0.11	0.62	24.85	-0.35	0.32	1.17	0.00
Hypothalamus-right	0.02	1.77	0.08	0.88	0.05	0.45	20.07	0.17	0.27	0.43	0.00
LateralGeniculum-left	0.70	0.14	0.01	0.98	-0.07	0.60	13.67	0.01	0.25	0.00	0.00
LateralGeniculum-right	0.43	0.36	0.03	0.97	-0.31	0.97	10.21	-0.36	0.25	2.04	0.00
LateralGlobusPallidus-left	0.00	10.72	0.22	0.38	-0.83	1.00	127.76	-1.15	1.01	1.29	0.01
LateralGlobusPallidus-right	0.00	11.36	0.22	0.36	-0.18	0.64	86.03	0.01	0.82	0.00	0.00
MammillaryBody-left	0.00	3.73	0.12	0.71	0.02	0.09	285.97	0.23	1.05	0.05	0.00

MammillaryBody-right	0.00	2.35	0.09	0.84	-0.03	0.15	308.34	-0.39	1.19	0.11	0.00
MedialGeniculum-left	0.39	0.40	0.03	0.97	-0.30	0.93	26.36	-0.61	0.32	3.56	0.01
MedialGeniculum-right	0.87	0.06	0.01	0.99	-0.27	0.93	30.66	-0.35	0.35	1.02	0.00
MedialGlobusPallidus-left	0.00	4.32	0.13	0.67	0.14	0.50	37.28	0.59	0.42	1.94	0.01
MedialGlobusPallidus-right	0.00	5.43	0.15	0.63	0.32	0.86	27.79	0.64	0.45	2.06	0.01
Pulvinar-left	0.00	3.95	0.12	0.69	0.09	0.42	144.89	0.28	0.85	0.11	0.00
Pulvinar-right	0.00	2.61	0.10	0.83	0.04	0.26	241.88	0.23	1.23	0.03	0.00
Putamen-left	0.00	6.49	0.16	0.58	0.00	0.03	664.95	-0.25	2.19	0.01	0.00
Putamen-right	0.00	7.99	0.18	0.54	0.00	0.09	336.12	0.04	1.54	0.00	0.00
RedNucleus-left	0.01	2.17	0.09	0.86	0.00	0.19	48.05	0.19	0.58	0.11	0.00
RedNucleus-right	0.13	0.86	0.05	0.94	-0.07	0.44	42.30	-0.34	0.50	0.46	0.00
SubstantiaNigra-left	0.78	0.10	0.01	0.99	-0.01	0.26	17.66	0.00	0.16	0.00	0.00
SubstantiaNigra-right	0.59	0.22	0.02	0.97	0.25	0.99	17.68	0.45	0.18	6.00	0.01
SubthalamicNucleus-left	0.10	0.96	0.05	0.94	-0.05	0.50	32.93	-0.18	0.38	0.21	0.00
SubthalamicNucleus-right	0.04	1.39	0.07	0.90	0.04	0.31	40.45	0.20	0.44	0.21	0.00
Thalamus_LateralDorsal-left	0.00	4.42	0.13	0.67	-0.02	0.13	97.75	-0.45	0.82	0.30	0.00
Thalamus_LateralDorsal-right	0.00	3.01	0.11	0.78	0.00	0.18	66.57	0.05	0.68	0.01	0.00
Thalamus_LateralPosterior-left	0.00	4.42	0.13	0.67	0.30	0.86	104.72	0.46	0.87	0.27	0.00
Thalamus_LateralPosterior-right	0.01	2.03	0.08	0.86	0.15	0.52	80.51	0.75	0.79	0.91	0.01
Thalamus_MedialDorsal-left	0.00	5.80	0.15	0.61	0.01	0.11	523.92	0.14	1.95	0.00	0.00
Thalamus_MedialDorsal-right	0.00	4.66	0.14	0.66	-0.01	0.10	658.68	-0.09	2.06	0.00	0.00
Thalamus_VentralAnterior-left	0.00	4.44	0.13	0.66	0.03	0.32	72.59	0.25	0.74	0.11	0.00
Thalamus_VentralAnterior-right	0.00	4.10	0.13	0.69	0.01	0.22	129.02	-0.15	0.95	0.02	0.00
Thalamus_VentralLateral-left	0.00	3.88	0.12	0.70	0.01	0.10	162.36	-0.13	1.09	0.01	0.00
Thalamus_VentralLateral-right	0.00	3.06	0.11	0.77	0.03	0.27	346.66	0.46	1.51	0.09	0.00
Thalamus_VentralPosteriorLateral-left	0.00	3.30	0.11	0.74	0.03	0.20	208.09	0.09	1.26	0.01	0.00
Thalamus_VentralPosteriorLateral-right	0.07	1.10	0.06	0.93	-0.04	0.20	237.74	-0.44	1.33	0.11	0.00
Thalamus_VentralPosteriorMedial-left	0.00	2.90	0.10	0.79	0.00	0.07	334.64	-0.01	1.46	0.00	0.00
Thalamus_VentralPosteriorMedial-right	0.07	1.10	0.06	0.92	-0.05	0.21	426.66	-0.27	1.48	0.03	0.00
Lt-angular_gyrus	0.00	14.28	0.25	0.28	-0.01	0.07	116.90	-0.42	0.89	0.23	0.00
Lt-caudate	0.65	0.18	0.01	0.98	-0.01	0.04	638.64	-0.01	2.02	0.00	0.00
Lt-cingulate_gyrus	0.00	19.11	0.29	0.12	-0.02	0.03	816.47	-0.81	2.33	0.12	0.01
Lt-cuneus	0.01	1.86	0.08	0.88	0.09	0.14	231.79	1.20	1.43	0.71	0.01
Lt-fusiform_gyrus	0.00	11.75	0.23	0.34	-0.03	0.10	134.82	-0.44	1.01	0.19	0.00
Lt-gyrusrectus	0.00	14.18	0.25	0.28	0.01	0.05	300.07	0.03	1.39	0.00	0.00
Lt-hippocampus	0.00	15.30	0.26	0.21	-0.04	0.13	370.59	-0.62	1.48	0.18	0.01
Lt-inferiorfrontal	0.00	25.29	0.35	0.00	-0.08	0.11	189.31	-1.03	1.01	1.03	0.01
Lt-inferioroccipital_gyrus	0.00	4.75	0.14	0.64	0.03	0.16	119.60	0.47	1.00	0.22	0.00
Lt-inferiortemporal_gyrus	0.00	13.76	0.25	0.29	0.01	0.05	239.45	0.11	1.04	0.01	0.00
Lt-insularcortex	0.00	19.25	0.29	0.11	0.29	0.84	543.03	0.62	1.94	0.10	0.01
Lt-lateralorbitofrontal_gyrus	0.00	23.42	0.33	0.02	0.68	0.98	193.74	1.41	1.19	1.40	0.02
Lt-lingual_gyrus	0.00	3.21	0.11	0.75	-0.20	0.37	264.70	-0.93	1.72	0.29	0.01
Lt-middlefrontal_gyrus	0.00	20.42	0.31	0.07	0.00	0.02	356.55	-0.10	1.39	0.01	0.00
Lt-middleoccipital_gyrus	0.00	10.02	0.21	0.43	0.19	0.54	260.34	0.81	1.70	0.23	0.01
Lt-middleorbitofrontal_gyrus	0.00	14.92	0.26	0.24	-0.48	0.82	190.16	-1.45	0.94	2.37	0.02
Lt-middletemporal_gyrus	0.00	19.00	0.29	0.13	-0.03	0.14	370.30	-0.26	1.23	0.04	0.00
Lt-parahippocampal_gyrus	0.00	13.80	0.25	0.29	0.03	0.08	219.14	0.63	1.34	0.22	0.01
Lt-postcentral_gyrus	0.00	8.47	0.19	0.52	0.00	0.05	185.67	0.30	1.20	0.06	0.00
Lt-precentral_gyrus	0.00	8.88	0.19	0.49	0.03	0.11	248.37	0.60	1.36	0.20	0.01
Lt-precuneus	0.00	4.14	0.13	0.69	-0.05	0.19	607.37	-0.65	2.08	0.10	0.01
Lt-putamen	0.04	1.35	0.06	0.91	0.10	0.87	3.62	0.11	0.21	0.26	0.00
Lt-superiorfrontal_gyrus	0.00	17.55	0.28	0.16	-0.30	0.37	473.30	-1.59	1.63	0.95	0.02
Lt-superioroccipital_gyrus	0.00	6.80	0.17	0.58	0.10	0.53	71.45	0.37	0.80	0.21	0.00
Lt-superiorparietal_gyrus	0.00	7.34	0.17	0.56	0.03	0.15	163.84	0.57	1.11	0.27	0.01
Lt-superiortemporal_gyrus	0.00	19.99	0.30	0.08	-0.01	0.06	442.86	-0.07	1.42	0.00	0.00
Lt-supramarginal_gyrus	0.00	13.66	0.24	0.29	0.04	0.14	161.64	0.33	1.02	0.10	0.00
Rt-angular_gyrus	0.00	13.36	0.24	0.30	0.07	0.18	201.89	0.42	1.12	0.14	0.00
Rt-caudate	0.18	0.73	0.04	0.96	0.00	0.04	455.03	-0.10	1.91	0.00	0.00
Rt-cingulate_gyrus	0.00	19.00	0.29	0.12	0.00	0.01	622.04	-0.10	2.02	0.00	0.00
Rt-cuneus	0.01	2.16	0.09	0.86	0.00	0.06	230.09	0.12	1.38	0.01	0.00
Rt-fusiform_gyrus	0.00	11.45	0.22	0.35	0.13	0.30	155.27	0.63	1.08	0.34	0.01
Rt-gyrusrectus	0.00	12.87	0.24	0.32	-0.18	0.26	303.52	-1.08	1.44	0.56	0.01
Rt-hippocampus	0.00	16.50	0.27	0.18	-0.02	0.08	265.78	-0.34	1.21	0.08	0.00
Rt-inferiorfrontal_gyrus	0.00	24.41	0.34	0.01	-0.01	0.06	208.50	-0.26	1.06	0.06	0.00
Rt-inferioroccipital_gyrus	0.00	3.06	0.11	0.77	-0.36	0.88	113.24	-0.91	1.00	0.84	0.01
Rt-inferiortemporal_gyrus	0.00	13.08	0.24	0.31	-0.04	0.14	234.27	-0.56	1.20	0.21	0.00
Rt-insularcortex	0.00	19.25	0.29	0.11	0.04	0.14	1073.48	0.33	2.78	0.01	0.00
Rt-lateralorbitofrontal_gyrus	0.00	22.32	0.32	0.03	0.05	0.27	346.41	0.45	1.33	0.11	0.00
Rt-lingual_gyrus	0.00	2.99	0.11	0.79	-0.11	0.35	286.29	-0.60	1.51	0.16	0.01

Rt-middlefrontal_gyrus	0.00	19.44	0.30	0.10	-0.14	0.31	402.75	-0.94	1.47	0.41	0.01
Rt-middleoccipital_gyrus	0.00	9.22	0.20	0.47	0.01	0.05	209.57	0.34	1.28	0.07	0.00
Rt-middleorbitofrontal_gyrus	0.00	16.04	0.27	0.19	0.00	0.07	166.62	0.01	0.95	0.00	0.00
Rt-middletemporal_gyrus	0.00	19.95	0.30	0.08	-0.30	0.46	568.87	-1.22	1.74	0.50	0.01
Rt-parahippocampal_gyrus	0.00	15.14	0.26	0.22	0.05	0.35	161.51	-0.06	1.10	0.00	0.00
Rt-postcentral_gyrus	0.00	5.19	0.14	0.63	-0.21	0.65	205.14	-0.63	1.24	0.26	0.01
Rt-precentral_gyrus	0.00	8.24	0.19	0.53	0.02	0.12	129.33	0.72	0.81	0.78	0.01
Rt-precuneus	0.00	3.58	0.12	0.72	-0.09	0.40	367.38	-0.37	1.71	0.05	0.00
Rt-putamen	0.03	1.51	0.07	0.90	0.00	0.04	573.50	-0.33	2.06	0.03	0.00
Rt-superiorfrontal_gyrus	0.00	17.34	0.28	0.16	-0.04	0.12	601.77	-0.55	1.93	0.08	0.01
Rt-superioroccipital_gyrus	0.00	5.92	0.16	0.61	0.17	0.44	145.50	0.83	0.96	0.76	0.01
Rt-superiorparietal_gyrus	0.00	5.19	0.14	0.63	0.02	0.11	137.73	0.63	1.06	0.35	0.01
Rt-superiortemporal_gyrus	0.00	22.95	0.33	0.02	0.01	0.02	410.36	0.01	1.65	0.00	0.00
Rt-supramarginal_gyrus	0.00	10.67	0.21	0.39	0.02	0.08	191.03	0.25	1.14	0.05	0.00
brainstem	0.00	3.77	0.12	0.71	-0.03	0.22	20.85	0.02	0.41	0.00	0.00
cerebellum	0.00	3.39	0.11	0.74	0.01	0.01	476.20	-0.23	1.94	0.01	0.00

Supplementary Table 2: Generalization Performance via Leave-One-Database-Out Validation

SVM	Study	AUC
SVM w/o COBRE	COBRE	**0.91967**
SVM w/o MCICShare		0.99795
SVM w/o NUSDAST		0.97586
SVM w/o BrainGluSchi		0.99850
SVM w/o NMorphCH		0.98513
SVM w/o COBRE	MCICShare	0.98262
SVM w/o MCICShare		**0.99942**
SVM w/o NUSDAST		0.97605
SVM w/o BrainGluSchi		0.99913
SVM w/o NMorphCH		0.99652
SVM w/o COBRE	NUSDAST	0.97133
SVM w/o MCICShare		0.99822
SVM w/o NUSDAST		**0.79286**
SVM w/o BrainGluSchi		0.99782
SVM w/o NMorphCH		0.98301
SVM w/o COBRE	BrainGluSchi	0.96370
SVM w/o MCICShare		0.99839
SVM w/o NUSDAST		0.96432
SVM w/o BrainGluSchi		**0.91641**
SVM w/o NMorphCH		0.98173
SVM w/o COBRE	NMorphCH	0.99257
SVM w/o MCICShare		0.99845
SVM w/o NUSDAST		0.98793
SVM w/o BrainGluSchi		0.99969
SVM w/o NMorphCH		**0.82229**
